# A systematic scientometric review of paternal inheritance of acquired metabolic traits

**DOI:** 10.1186/s12915-023-01744-6

**Published:** 2023-11-13

**Authors:** Luís Crisóstomo, Pedro F. Oliveira, Marco G. Alves

**Affiliations:** 1 Departmento de Anatomia, UMIB - Unidade Multidisciplinar de Investigação Biomédica, ICBAS - Instituto de Ciências Biomédicas Abel Salazar, Universidade Do Porto, Rua de Jorge Viterbo Ferreira 228, 4050-313 Porto, Portugal; 2 Laboratory for Integrative and Translational Research in Population Health (ITR), University of Porto, Porto, Portugal; 3 MediCity Research Laboratory, University of Turku, Turku, Finland; 4 LAQV-REQUIMTE and Department of Chemistry, University of Aveiro, Campus Universitário de Santiago, Aveiro, Portugal; 5 Biotechnology of Animal and Human Reproduction (TechnoSperm), Institute of Food and Agricultural Technology, University of Girona, Girona, Spain; 6 Unit of Cell Biology, Department of Biology, Faculty of Sciences, University of Girona, Girona, Spain; 7 Institute of Biomedicine - iBiMED and Department of Medical Sciences, University of Aveiro, 3810-193 Aveiro, Portugal

**Keywords:** Translational research, Epigenetic inheritance, Systematic review, Multiomics, Scientometrics

## Abstract

**Background:**

The concept of the inheritance of acquired traits, a foundational principle of Lamarck’s evolutionary theory, has garnered renewed attention in recent years. Evidence for this phenomenon remained limited for decades but gained prominence with the Överkalix cohort study in 2002. This study revealed a link between cardiovascular disease incidence and the food availability experienced by individuals’ grandparents during their slow growth periods, reigniting interest in the inheritance of acquired traits, particularly in the context of non-communicable diseases. This scientometric analysis and systematic review comprehensively explores the current landscape of paternally transmitted acquired metabolic traits.

**Results:**

Utilizing Scopus Advanced search and meticulous screening, we included mammalian studies that document the inheritance or modification of metabolic traits in subsequent generations of unexposed descendants. Our inclusive criteria encompass intergenerational and transgenerational studies, as well as multigenerational exposures. Predominantly, this field has been driven by a select group of researchers, potentially shaping the design and focus of existing studies. Consequently, the literature primarily comprises transgenerational rodent investigations into the effects of ancestral exposure to environmental pollutants on sperm DNA methylation. The complexity and volume of data often lead to multiple or redundant publications. This practice, while understandable, may obscure the true extent of the impact of ancestral exposures on the health of non-exposed descendants. In addition to DNA methylation, studies have illuminated the role of sperm RNAs and histone marks in paternally acquired metabolic disorders, expanding our understanding of the mechanisms underlying epigenetic inheritance.

**Conclusions:**

This review serves as a comprehensive resource, shedding light on the current state of research in this critical area of science, and underscores the need for continued exploration to uncover the full spectrum of paternally mediated metabolic inheritance.

**Supplementary Information:**

The online version contains supplementary material available at 10.1186/s12915-023-01744-6.

## Background

The publication of the results of the Human Genome Project in the first years of the twenty-first century [1] rose more questions than answers about the “building blocks of life”. It became evident that the information encoded in the human genome could not explain all the spectra of phenotypes observed and account for the majority of risks for diseases [2]. Just a year before the publication of those results, a paper by a group of Swedish researchers [3] challenged the objectives of the Human Genome Project. This paper suggested that environmental factors, particularly food availability, were associated with increased risk for the onset of cardiovascular diseases after two generations, in an isolated human community. Although this paper does not mention any mechanism of epigenetic inheritance, the results promoted the discussion whether epigenetic inheritance could occur in humans.

Epigenetic inheritance resumes some of the empirical principles of the evolution theory suggested by Lamarck in the nineteenth century. Notably, both share the key principle that an individual acquires traits over its lifespan that are later transmitted to the offspring. Despite the popularity of the “Survival of the fittest” postulated by Darwin, especially after the works of Mendel on the inheritance of selected characteristics, Lamarck’s principles were never completely abandoned. Conrad Hal Waddington pioneered in the study of epigenetics, a term often attributed to himself [4, 5]. His works set the basis for modern epigenetics, even before DNA was described as the source of genetic information in living systems. Over the course of his scientific career, he developed the concept of “epigenetic landscape in the course of time” [6, 7], a representation of the interaction between genome and environment (via epigenome) to create the phenotype.

**Fig. 1:**
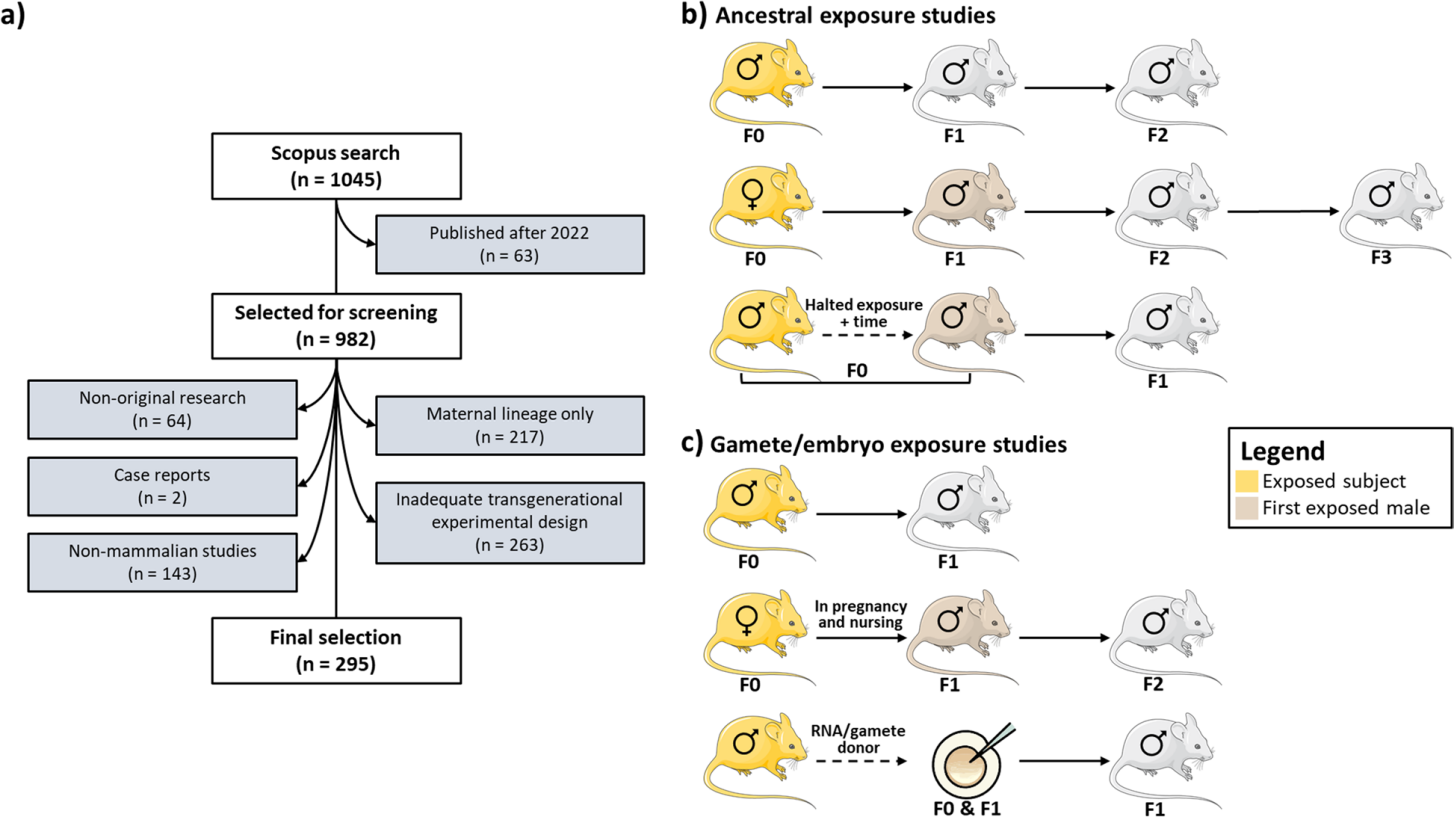
Paper selection and categorization criteria. **a** Flow-chart of the literature search. **b** Illustration of experimental designs categorized as “Ancestral exposure studies”. **c** Illustration of experimental designs categorized as “Gamete/embryo exposure studies”. Multigenerational studies follow one of these experimental studies, but the exposure is repeated in more than one generation

Biomedical research in epigenetic inheritance has gained momentum over the last years, as non-communicable diseases become the leading cause or premature death and incapacity in the world [8]. Despite the early evidence of the presence of active DNA polymerase [9] and specific RNA transcripts [10] in human spermatozoa, it was widely accepted that spermatozoa were transcriptionally silent. It was just in 1997 that Wykes and colleagues demonstrated the expression of RNA transcripts in mature spermatozoa, and at a similar level of expression as reported in testes [11]. Nevertheless, the role of paternal epigenetic inheritance in the phenotype of progeny was overshadowed by the matrilinear epigenetic inheritance. For that contributed the description of the molecular mechanisms of the maternal inheritance of the *agouti* phenotype in mice [12] and the lack of known epigenetic transfer mechanisms via the male and the offspring [13]. As more studies in epigenetic inheritance were published, lifestyle and environmental exposures have gained more relevance in disease risk, onset, and prevention. Thus, the number of papers on paternal epigenetic inheritance of metabolic traits has also increased. This has created the need to systematize the current knowledge and identify trends and gaps in the published studies. The state of the art on this subject was recently reviewed by Xu et al*.* [14], but the authors have not critically discussed the main results of the studies in the context of research trends and practices. Our comprehensive systematic review provides a thorough analysis of current knowledge pertaining to the paternal inheritance of acquired metabolic traits. To accomplish this, we undertook a scientometric study encompassing all research investigations documenting the paternal inheritance of acquired metabolic traits in mammals, including humans (Fig. 1). Our inclusive approach covers both descriptive studies, which solely document the inheritance of phenotypes, as well as mechanistic studies that delve into the epigenetic mechanisms underlying paternal inheritance. We delve into the primary strengths of the various experimental settings employed by researchers to investigate the involvement of males in the transmission of acquired traits. Additionally, we explore the hurdles encountered by authors conducting studies on paternal epigenetic inheritance, stemming from the constraints imposed by publishing policies. These artificial limits in the “Digital Era” will create a challenge to future meta-analyses on the subject. As an example, we conduct multiomics integration using data from selected publications obtained from the same animal experiment, to demonstrate how these practices may negatively affect an integrative view of the mechanisms involved in the paternal (epigenetic) inheritance of metabolic traits.

## Scientometric characterization of epigenetic inheritance studies

**Fig. 2:**
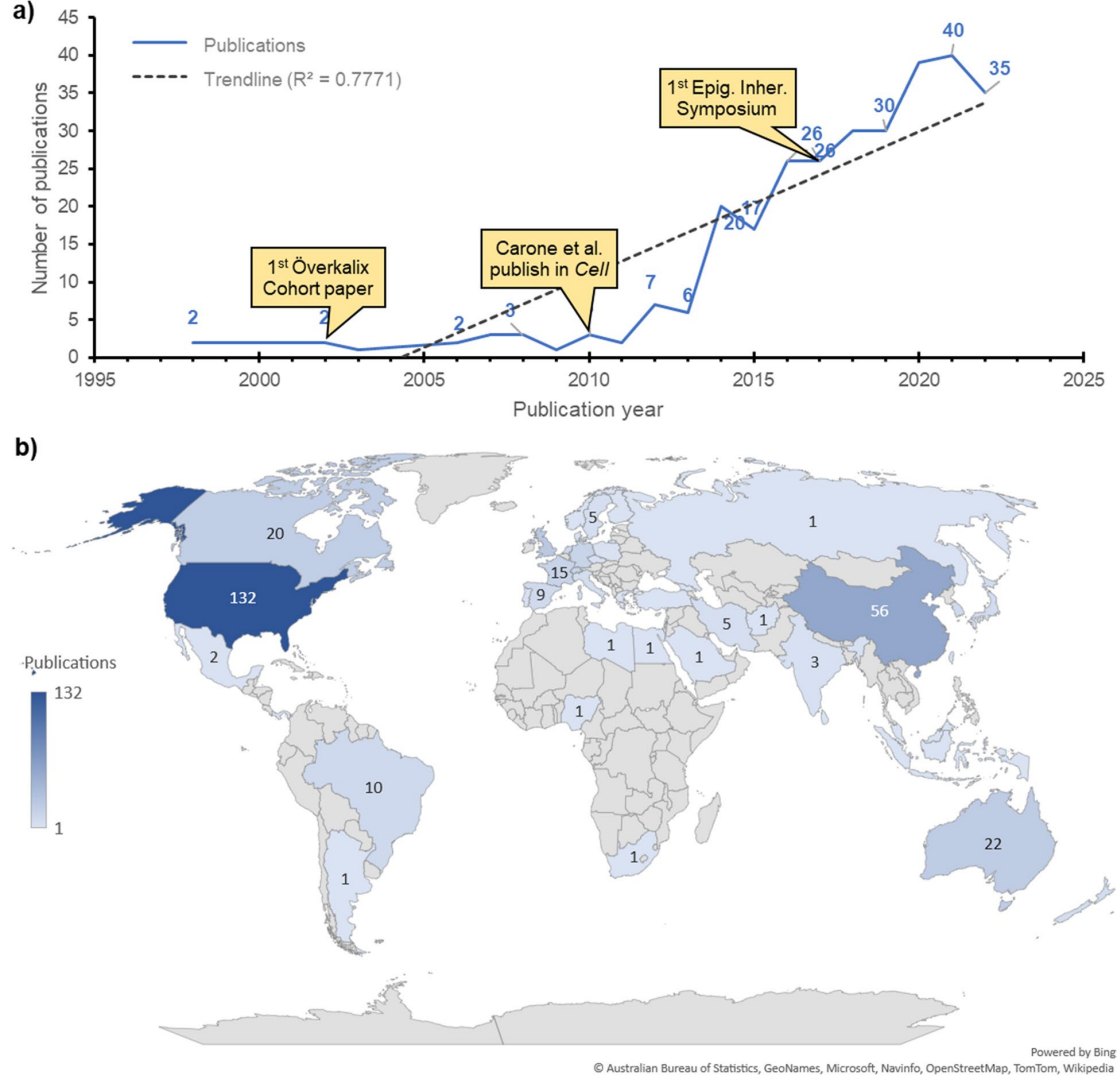
Publication trends in paternal epigenetic inheritance. **a** The number of publications has increased at a 1.9 papers/year rate (*r*^2^ = 0.777). Until 2011, few studies on the subject were published, although the 1st Överkalix cohort publication had been published in 2002. In 2010, Carone and colleagues published an epigenetic inheritance paper in *Cell*, which may have contributed to increase the visibility of the subject. After that, the number of publications per year increased significantly until 2021. **b** The main origin of publications on epigenetic inheritance is the USA, which have produced more than twice as many papers on the subject than the second most prolific country, China. The UK, Australia, and Canada follow, completing the top-5 countries in terms of publications

The first papers on the epigenetic inheritance of metabolic traits via the paternal lineage date back to 1998 [15, 16]. The number of publications per year has been increasing steadily at a 1.9 papers/year rate (*r*^2^ = 0.771) although it did not increase significantly until 2011 (Fig. 2a). Interestingly, 5 of the 10 most cited papers in the subject have been published during this period [3, 17–20], including the first paper of the Överkalix cohort study [3] (Additional file 1, table S1). The fast increase in publications about paternal epigenetic inheritance of metabolic traits after 2011 may be related to the first-ever publication of an ancestral exposure, descriptive (associated) study in Cell [17], which is also the most cited paper in paternal epigenetic inheritance of metabolic traits. Besides, some of the ongoing epigenetics meetings and symposia trace back to 2010/11, such is the case of “Epigenetic Focus” (Max Planck Institute of Immunobiology and Epigenetics, Germany, 2010) and the “Epigenetics Eh!” (Ontario, Canada, 2011) [21].

**Fig. 3:**
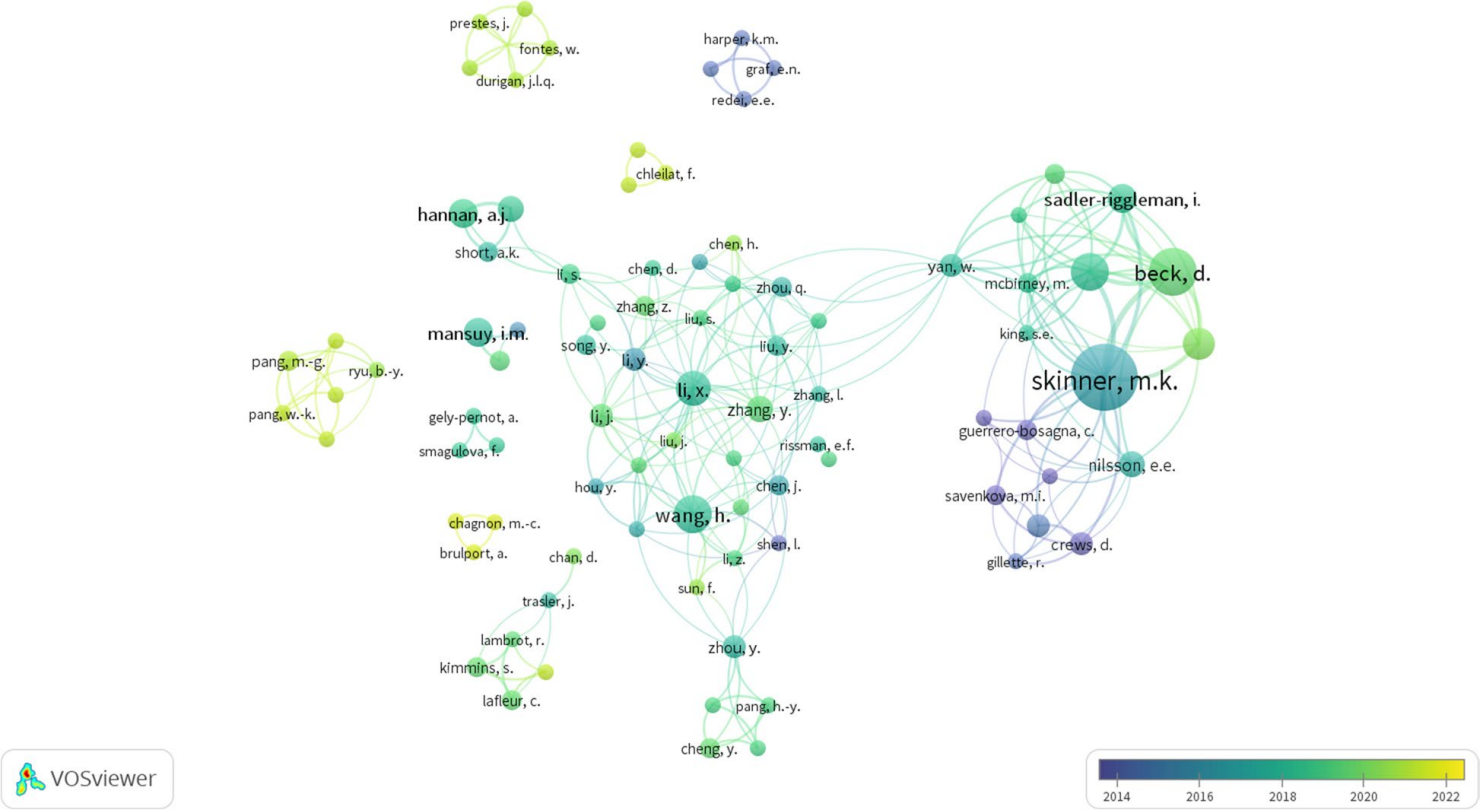
Authorship networks in paternal epigenetic inheritance of metabolic traits. Node size is proportional to the number of publications while the color gradient represents the mean year of publications. Authors with three or less publications and less than two links to other authors were omitted. M.K. Skinner at the Washington State University Pullman (US) is the leading author in terms of total number of papers on the subjects (23 publications, supplementary table S2). Six of the top ten authors according to number of publications are affiliated with the Washington State University Pullman (US), which is the leading institution using the same metric (24 publications, Table 1). This close collaboration is clear in the figure, with M.K. Skinner as the central node. Besides, the figure clearly shows other relevant research nuclei at the Shanghai Jiao Tong University (China) and at The Florey (Australia), which are now interconnected with the Washington State University Pullman by co-authorships

The United States lead the total number of publications on the subject, with more than double the number of publications of the following country, RP of China (Fig. 2b). However, this gap has been closing in the past 5 years. Consequently, the list of institutions (Table 1) and authors (Additional file 1, table S2) is led by US-based institutes and authors. Particularly, the research group led by Skinner M.K. at the Washington State University Pullman has authored about 8% of all publications in paternal transgenerational inheritance of metabolic traits. Six out of the ten most productive researchers in the subject belong to this research group (Additional file 1, table S2). The second most relevant research clusters, considering number of publications, are the Shanghai Jiao Tong University (School of Medicine), in the PR of China and the University of Melbourne, in Australia (Table 1). Interestingly, according to the data extracted from SCOPUS, the leading labs on the topic have already established scientific collaborations (Fig. 3). Besides this large cluster of researchers, our analysis shows eight other relevant research nuclei located in Europe (Inserm, France and Universität Zürich/ETH Zürich, Switzerland), in the USA (Northwestern University, Chicago), in Canada (McGill University and University of Calgary), and in PR of China (Zhejiang University).

**Table 1:** Top 15 ranked Institutions according to the number of papers published in paternal Transgenerational Epigenetic Inheritance in mammalians before 2023 (Updated in August 2023)

Rank	Institution	Country	Publications
**1**	Washington State University Pullman	United States	24
**2**	Shanghai Jiao Tong University (School of Medicine)	China	11
	University of Melbourne	Australia	11
**4**	Inserm	France	9
	Ministry of Education of the People's Republic of China	China	9
	Université McGill	Canada	9
**7**	University of Pennsylvania	United States	8
	Universität Zürich	Switzerland	8
**9**	Harvard Medical School	United States	7
	University of California, Irvine	United States	7
	The University of Texas at Austin	United States	7
	ETH Zürich	Switzerland	7
	The Florey	United Kingdom	7
	International Peace Maternity & Child Health Hospital of China welfare institute	China	7
**15**	University of Nevada, Reno	United States	6

Notwithstanding these trends, it is noteworthy that the journal at the forefront of publishing the most studies on this subject is *Scientific Reports*, based in the UK (Table 2). This generalist open access journal of the *Nature* group has published nearly 8% of all papers in our selection (23/295), followed by the US-based, generalist open access PLoS One (16/295). Among the top-10 journals according to the number of publications in paternal epigenetic inheritance, just two have more than 5 Impact Factor (IF) index – PNAS: Proceedings of the National Academy of Sciences of The United States of America (IF: 11.1) and the International Journal Of Molecular Sciences (IF: 5.6), as of 2023. There are only three journals specialized in Epigenetics among the top-10 journals featuring papers on this subject. Notably, one of these epigenetics journals is dedicated to Epigenetic Inheritance and received an IF for the first time in the latest edition of the JCR. Most of the studies on the subject are descriptive (association) studies (Table 3); therefore, they might be out of scope of specialized Epigenetics journals seeking for epigenetic mechanisms. Also, multidisciplinary journals might be more attractive to researchers to reach a broader range of readers and higher IFs.

**Table 2:** Top 10 ranked Journals according to the number of papers published in paternal Transgenerational Epigenetic Inheritance of metabolic traits in mammalians before 2023 (Updated August 2023). Generalist, multidisciplinary journals publish the most in the subject

Rank	Journal	Country	Journal IF (2022)^a^	Quartile (subject)^a^	Number of publications
**1**	Scientific Reports	United Kingdom	4.6	Q2	23
**2**	PLoS One	United States	3.7	Q2	16
**3**	Epigenetics	United States	3.7	Q3	7
**4**	Proceedings of the National Academy of Sciences of The United States of America	United States	11.1	Q1	7
**5**	Environmental Epigenetics	United Kingdom	3.8	Q2	6
	Epigenetics And Chromatin	United Kingdom	3.9	Q1	6
**7**	Biology Of Reproduction	United States	3.6	Q2	5
	Endocrinology	United States	4.8	Q2	5
	FASEB Journal	United States	4.8	Q1	5
	International Journal Of Molecular Sciences	Switzerland	5.6	Q1	5

^a^Data obtained via InCites Journal Citation Reports (Clarivate). For journals indexed to more than one subject, the highest quartile is considered

**Table 3:** Quantitative description of the manuscripts included in the systematic review (updated August 2023)

			**Exposure**							**Vehicle**	**Outcome**								
Study design	**Pub**	**Multi**	**Tox**	**Diet**	**Exe**	**Psy**	**sncRNA**	**KO/KI**	**Other**	**Sperm**	**Pheno**	**Behav**	**Metab**	**Microb**	**Endoc**	**sncRNA**	**lncRNA**	**Methyl**	**Histone**
Human, Prospective	17	4	3	3	0	1	0	0	9	0	10	2	2	0	1	0	0	2	0
Human, Retrospective (historical)	8	1	3	2	0	0	0	0	3	0	7	0	3	0	1	0	0	0	0
Animal, Ancestral exposure, Descriptive (associative)	105	9	45	29	8	15	1	4	10	30	71	40	77	5	20	13	1	39	8
Animal, Ancestral exposure, Mechanistic	15	2	3	3	0	2	2	6	5	12	4	2	6	0	0	5	0	10	4
Animal, Gamete/embryo exposure, Descriptive (associative)	130	75	82	33	5	10	0	0	10	33	90	29	102	2	31	10	0	49	15
Animal, Gamete/embryo exposure, Mechanistic	20	6	2	4	0	2	7	4	4	10	12	5	11	0	0	3	0	8	6

*Abbreviations*: *Pu* publications, *Multi* Multigenerational studies (i.e., studies on exposures repeated over multiple generations), *tox* toxicant, *Exe* exercise, *psy* psychological/mental challenge, *sncRNA* manipulation with exogenous small RNA, *KO/KI* gene knock-out/knock-in, *Pheno* phenotype, *Behav* behavior, *Metab* metabolic phenotype, *Microb* gut microbiota, *Endoc* Endocrine balance, *sncRNA* small non-coding RNA, *lncRNA* long non-coding RNA, *Methyl* methylation

## Study characteristics

### Human studies

Human studies provide the highest degree of experimental evidence of risk factors to human health, but their application is complex. Among the main challenges for conducting a reliable human study are the control of confounders, the definition and recruitment of adequate control groups, missing data, and the definition of exposure/intervention [22, 23]. When it comes to epigenetic inheritance studies, the quality of evidence is constrained by several factors, including the time lag between exposure and outcome, the presence of Nature vs. Nurture confounders, and the challenges in accurately measuring a before-and-after effect. Considering The Cambridge Quality Checklists [24] Causal Risk Factor Score, no epigenetic inheritance study could achieve more than 5 out of 7 points, precisely because it is not possible to measure “change” before and after exposure.

Human cohort studies represent 8.5% (25/295) of the publications meeting the selection criteria (Table 3). Most of the selected publications (18/25) score 5 in the Causal Risk Factor score, i.e., the experimental setting includes adequate controls and the results are corrected for confounding factors, but no analysis of change is performed. Also, nearly 60% of the selected studies score the highest in the Risk Factor score (14/25), indicating prospective data or the study of an unchangeable risk factor [24]. In our evaluation, we have granted the maximum score of 3 if the study is based in medical registries of the ancestors clearly stating the ancestral exposure to the risk factor in study. If this exposure could not be traced back to preconception, then the study scored 2 for Risk Factor. Prospective studies linking present-day outcomes in fathers and children, but without any indication of preconceptional or past exposure to the risk factor were scored 1. The correlates score was the most variable among selected publications (Table S3), as it integrates various elements of data collection such as randomization, sample size, and response rates.

The highest overall Cambridge Checklist score observed was 12. This score was achieved by two studies: a British prospective study based on two birth cohorts (NCDS and BCS70) associating paternal smoking with lower prevalence of type 1 diabetes in the offspring [25]; and a retrospective (historical) study from PR of China studying the effects of the Chinese Famine in several cardiovascular and morphological indexes of the descendants [26]. Multiple publications retrieved by our bibliography search algorithm are part of larger retrospective human cohort studies. Among the most notable cohorts are The Dutch Famine Birth Cohort [19, 27], the Great Chinese Famine cohort [26, 28], and the Swedish Överkalix Cohort [3]. The Dutch Famine Birth Cohort includes birth records of term singletons born in the Wilhelmina Gasthuis (Amsterdam) between November 1943 and February 1947. These individuals were directly (in utero) or indirectly (preconception) exposed to the 1944–1945 Hongerwinter, a period of food supply shortage caused by a German blockage during World War II. The Great Chinese Famine cohort shares key characteristics with the Dutch Famine Birth Cohort, as both investigate the consequences of undernutrition. Specifically, the Great Chinese Famine cohort comprises individuals born during the Great Chinese Famine (1959–1961) or within 5 years before and after this period. This cohort has been integral to studies exploring intergenerational and transgenerational inheritance, leveraging data from the China Health and Nutrition Survey. One of the most renowned investigations into transgenerational inheritance in a human population is the Överkalix Cohort study. This study encompasses three distinct birth cohorts originating from the small northern Swedish parish of Överkalix, with births occurring 15 years apart (1890, 1905, and 1920). Through a meticulous analysis of historical, agricultural, and socioeconomic records, researchers have been able to estimate food availability during periods of slow population growth and, consequently, discern the lasting health effects on subsequent generations. Other cohorts include the MAGIC cohort (UK) [29], the SHARE cohort (US) [30], the Michigan PBB Registry (US) [31], the ALSPAC cohort (UK) [32, 33], the LifeLines cohort (Netherlands) [34], and the Integrated Screening Program (Taiwan) [35, 36].

The number of human studies is limited compared to the number of studies on epigenetic inheritance of metabolic traits conducted in animals. However, this number is expected to increase in the near future, as many countries have initiated large longitudinal transgenerational studies to better understand the cumulative effects of lifestyle choices. Some examples are found in other Nordic countries, such as the Young Finns Cohort and the Norwegian Mother, Father and Child Cohort Study (MoBa).

### Animal studies

#### Ancestral exposure studies

Ancestral exposure models involve the exposition of a subject to a stimulus (such as diet, chemicals, physical activity) and subsequently investigating the effects of this exposure in offspring who were not directly exposed to the initial stimulus. If the effect is measured in the grandoffspring and the offspring has not been exposed, then the study reports “transgenerational effects” (grandfather-son-grandson and onwards). When the impact of the exposure is observed or measured in the offspring, the study reports “intergenerational effects” specifically referring to the transmission from one generation to the next (e.g., father to son). However, it is worth noting that intergenerational studies of ancestral exposure are relatively uncommon due to the necessity of discontinuing the exposure before the conception of the subsequent generation. The experimental designs admitted as Ancestral Exposure studies are depicted in Fig. 1b. Two fifths of the publications included in this systematic review fall within this category (40.7%, 120/295) (Table 3).

The most cited paper on paternal epigenetic inheritance of metabolic traits is precisely an ancestral exposure, descriptive (associative) study associating the intergenerational effects of low-protein diet in hepatic gene expression of non-exposed offspring [17]. This study provided one of the earliest experimental evidence of paternal inheritance of traits via an epigenetic mechanism (i.e., DNA methylation). The earliest evidence of paternal epigenetic inheritance of metabolic traits via sperm miRNAs was obtained using a model of Ancestral Exposure [37]. In this study, Fullston et al. [37] demonstrated that excess adiposity caused by HFD altered sperm miRNA content and germ cell methylation status that persisted up to two generations downstream the exposure. Despite those notable examples, two fifths of the publications based on ancestral exposure models refer to studies on the trans- and intergenerational effects of the exposure to environmental toxicants (48/120, 40%). Among those studies are several publications authored by the research group of M.K. Skinner, the most prolific author in paternal epigenetic inheritance [38–42].

#### Gamete/embryo exposure studies

In gamete/embryo exposure models, embryos or gametes are exposed to stimuli in the external media or by intracytoplasmic injection, and the effects of this exposure are evaluated in the viable subject generated from the exposed embryo or gamete. This includes studies of intergenerational paternal inheritance where the male progenitor is exposed to the stimulus in the peri-conceptional period. In this systematic review, we have also categorized models of in utero exposure as gamete/embryo exposure, since when the pregnant female is exposed to stimuli, ultimately the embryo is equally exposed. The experimental designs classified as gamete/embryo exposure studies in this systematic review are illustrated in Fig. 1c. More than half of the publications on paternal epigenetic inheritance included in this review describe gamete/embryo exposures (50.8%, 150/295) (Table 3). These studies may report intergenerational (mother-son-grandson or father-son) or transgenerational effects (mother – son – grandson – grand-grandson and onwards). Almost all intergenerational inheritance studies are a gamete/embryo exposure design. This experimental setting has great potential for the study of mechanisms of epigenetic inheritance, particularly via sncRNA. The second and the third most cited publications included in this systematic review are both intergeneration gamete exposure study describing the acquisition of traits via sncRNA [43, 44] (Additional file 1, table S1). Both studies have used a similar strategy; male mice were exposed to stimuli (trauma and HFD, respectively) and then sperm RNAs from the exposed mice were extracted and injected into unexposed zygotes. In both studies, the authors observed the same traits observed in the exposed mice.

Similar to ancestral exposure studies, more than half of the publications based on gamete/embryo exposure models focus on the effects of exposure to environmental toxicants (56%, 84/150). Again, a major contribution for this trend is due to the research group of M. K. Skinner at the Washington State University Pullman [20, 45–57]. The vast majority of the in utero exposure studies were categorized as gamete/embryo exposure studies. The exception are maternal inheritance studies where the exposure was withdrawn once pregnancy was confirmed and if outcomes are measured in the grand-grandoffspring of the exposed female. However, in utero exposure studies were not categorized and counted together as the focus of the systematic review is patrilinear inheritance of metabolic traits.

#### Multigenerational exposure studies

Multigenerational exposure studies usually include initial exposure, offspring exposure, and outcome exposure. These studies follow any of the experimental settings compatible with ancestral and gamete/embryo exposure (Fig. 1b, c), with the difference that the exposure is repeated in more than one generation. Follow-up exposures may occur in multiple generations except the generation where the outcome is measured, but the response to the same exposure may be an outcome itself. Therefore, multigenerational studies allow the simulation of complex human exposures in a controlled environment and shorter time, but they require multiple control groups to attempt to isolate causal relations between the different exposures and their outcomes. In our selection, multigenerational exposure studies represent 25.1% (74/295) of the publications in paternal epigenetic inheritance of metabolic traits (Table 3).

None of the top-10 most cited publications in paternal epigenetic inheritance of metabolic traits adopts a multigenerational design. Yet, this experimental design is the most recent: the first paper implementing a multigenerational design dates back to 2007 [58]. This pioneer study aimed at studying the hepatic effects of in utero exposure to two xenoestrogens in drinking water for 3 generations, using rats. The authors report an increase in the number of apoptotic and binuclear hepatocytes, and accumulation of γ-glycogen in liver of exposed animals of the third generation. However, the cumulative risk for the observed phenotypes is not discussed under the light of epigenetic inheritance. This model of multigenerational exposure to environmental toxicants may have caused impact among the researchers in the field of environmental exposure. Three years later, the second paper adopting a multigenerational model retrieved by our bibliographical search was published by Guerrero-Bosagna et al. [20]. This paper adopted a complex multigenerational model of gamete/embryo exposure to the toxicant vinclozolin. Pregnant mice were exposed to vinclozolin to generate the first exposed male generation (M-F1) and the first and second exposed female generation (F-F1 and F-F2). Since female embryos already carry gametes arrested after the first mitotic division, in utero exposure affects two female generations. Several litters were generated, and mice from different litters but from the same generation were mated together to generate the next generation (e.g., F-F1 x M-F1, F-F2 x M-F2). F2 males were mated with F2 females, whose oocytes were exposed to vinclozolin in utero, thus introducing the second exposure to vinclozolin in generation F3. The outcomes of gamete/embryo exposure to vinclozolin were then measured in generation F3, by comparison F3 litters of lineages exposed to vinclozolin against unexposed F3 litters. This research group adopted a multigenerational model in 19 of their 23 publications (Table S2).

Multigenerational studies have also been used to study the cumulative effects of high-fat diet (HFD) and psychological trauma. A study by Raad et al. [59] combined multigenerational exposure to Western diet (a HFD during 5 generations) and intracytoplasmic injection of exogenous RNA extracted from diet-expose mice in naïve murine zygotes. Using this approach, the authors looked for an association between the duration of exposure and the severity of the phenotype. Interestingly, mice resulting from the manipulated zygotes did not acquire the obesity-related phenotype, contrary to previous studies using exogenous sncRNAs [43, 44]. Another study based on injection of exogenous sncRNA into the zygote investigated the effects of fear conditioning over 3 generations of mice [60]. The researchers isolated sperm sncRNAs from fear-conditioned mice by chronic exposure to the odor of a predator. The isolated sncRNA was injected into naïve zygotes and the generated mice from those zygotes were exposed to the same odor as the sncRNA donor upon reaching adulthood. Then males were mated with naïve females to generate the F2 generation, which underwent a fear conditioning protocol consisting of predator odor and shock. Interestingly, F2 males did show enhanced consolidation to this fear conditioning program, suggesting that the presence of the predatory odor over generations inhibited the fear response to this stimulus in F2 males [60]. In conclusion, multigenerational exposure studies offer a unique perspective on the complex interplay between environmental exposures and epigenetic inheritance of metabolic traits. These investigations encompass a series of exposures spanning multiple generations, providing insights into the cumulative effects of various stimuli within a controlled environment and have opened new avenues for investigating the cumulative risk and inheritance patterns associated with HFD, psychological trauma, and other environmental factors. Multigenerational exposure studies thus hold promise for unraveling the intricate mechanisms underlying paternal epigenetic inheritance of metabolic traits.

## Exposure characteristics

### Environmental toxicants

Nearly half of all the publications included in this systematic review describe the effects of environmental toxicants (46.7%, 138/295). This exposure may be confined to a single generation, to the embryonic and postnatal period, or even over the course of multiple generations. Globally, these works have focused on the effects of exposure to environmental toxicants [38–41, 61–69], to prescription drugs [70–74] and to recreational drugs [75–80]. The later raises special interest due to the prevalence of smoking and alcohol consumption, and recent discussions to liberalize cannabis consumption.

The effects of environmental toxicants on human health are also a topic of interest for researchers in Epidemiology and Occupational Health. As such, 24% (6/25) of all human studies included in this review describe the effects of toxicant exposures in human populations. One of these papers is a controversial paper conducted in the UK associating paternal smoking with the prevalence of type 1 diabetes in the descendants [25]. Surprisingly, the authors found lower incidence of type 1 diabetes in children from parents who smoke, compared to children of non-smoking parents. However, this result may be explained by the low prevalence of type 1 diabetes in both cohorts (0.7 and 0.4%), which also highlights the biases introduced by systematic scoring schemes of scientific papers. Despite the large cohort (more than 11,000 children), response and retention rates ≥ 70% and differential attrition ≤ 10%, the authors estimate a 0.44 odds ratio for the onset of type 1 diabetes in children of smoking fathers at 5 and 10 years of age. This observation does not exclude the possible onset of the disease later in life, although the peak onset age for type 1 diabetes is estimated at 5–7 years old [81].

The most prolific research group dedicated to the study of paternal epigenetic inheritance after in utero exposure to environmental toxicants is led by Michael K. Skinner [20, 38–42, 46–50, 53–57]. Taken together, this team’s papers provide an integrative overview of the paternally inherited phenotypes and epigenetic changes related to the exposure to some pollutants. The authors have demonstrated that in utero exposure to DDT promotes the onset of kidney and prostate disease, and the development of tumors up to the grand-grandchildren of exposed dams and via the paternal lineage [47, 48, 50, 54]. Besides those phenotypic differences, the authors report differences in sncRNA content, methylation rate, methylated regions, histone retention, and histone methylation in sperm and testis of descendants from in utero exposed males comparing to unexposed males. Similar effects were reported in descendants of males exposed to vinclozolin during gestation [20, 46–49, 53, 55–57]. Notably, the authors also report behavioral effects in the offspring of exposed mice. These effects include anxiety-like behavior linked to alterations in brain transcriptome and changes in mate preference, which are correlated with shifts in sperm RNA content. They also report increased adiposity and changes in age of puberty onset, which were associated with differently methylated regions of sperm DNA.

Studies on the exposure to drugs of abuse and alcohol were also categorized as “studies of exposure to environmental toxicants”. Among these publications, a study of alcohol exposure and stress response in the unexposed offspring is particularly innovative [82]. In this paper, the exposure to alcohol was indirect, via alcohol vapor, instead of the most typical direct alcohol intake. As alcohol is a physiological stressor, with this experimental setting, the authors sought to isolate the stress response from the ethanol toxicity. Adult animals were exposed to chronic ethanol vapor for 6 weeks, then mice mated with naïve females. Stress response and alcohol preference after restraint were evaluated in adult offspring. Interestingly, no differences in alcohol preference were found between the offspring of alcohol-exposed and unexposed mice, but the offspring of ethanol exposed mice had lower serum cortisol and higher fluid intake. Concerning drug abuse, Pachenari and colleagues [83] examined the transgenerational consequences of paternal morphine exposure during adolescence on pain perception and the antinociceptive effects of morphine in rat offspring. Male rats were administered increasing doses of morphine for 10 days during postnatal days 31–40. After a 20-day period post-morphine exposure, these male rats were mated with female rats, and behavioral tests were conducted on the resulting male offspring on postnatal day 60. The results revealed notable differences in pain-related behaviors between offspring of morphine-exposed and saline-exposed fathers, indicating a significant transgenerational effect of paternal morphine exposure on pain perception in rat offspring. Other study investigated the effects of adolescent male rat exposure to morphine on subsequent generations’ self-administration behaviors involving cocaine and opioids [84]. Male rats were exposed to increasing doses of morphine during adolescence and then mated with drug-naïve females in adulthood to produce F1 offspring. Results revealed that both male and female F1 rats exhibited delayed acquisition and reduced intake of cocaine, along with diminished effort compared to control rats. Female F1 rats also showed increased morphine intake and effort for oxycodone. Notably, even after acquiring morphine self-administration, both male and female F1 rats exhibited reduced interest in cocaine. The study identified altered histone acetylation and increased BDNF mRNA levels in specific brain regions as potential mechanisms underlying these generational effects of opioid exposure [84]. Another relevant environmental toxicant group is endocrine disruptors. The first paper implementing a multigenerational exposure model investigated the exposure to two xenoestrogens in rats [58]. In this study, xenoestrogens were dispensed in drinking water for three generations of male rats, and the effects of this chronic exposure were evaluated in liver.

There is a substantial impact of environmental toxicants, including prescription and recreational drugs, on paternal epigenetic inheritance. Research in this area has revealed transgenerational effects on various phenotypes, from kidney and prostate diseases to behavioral changes and stress responses. Furthermore, studies have demonstrated the complex interplay between epigenetic changes, RNA content, histone modifications, and DNA methylation, providing valuable insights into the mechanisms underlying these transgenerational effects. Additionally, studies suggest transgenerational consequences of drugs of abuse and alcohol exposure revealing significant alterations in pain perception, drug self-administration behaviors, and neurobiological mechanisms. Lastly, investigations into endocrine disruptors have also made valuable contributions to understanding multigenerational exposure models, particularly in the context of liver health. Overall, there is urgency of continued research into environmental toxicants and their far-reaching consequences, emphasizing the importance of considering both direct and multigenerational effects in understanding their impact on paternal epigenetic inheritance.

### Diet and microbiota

Diet ranks as the second most extensively studied exposure in publications related to paternal inheritance of metabolic traits, accounting for 25.1% of the total (74 out of 295). Notably, among these studies are some of the most prominent ones conducted in human cohorts. The publications derived from the Dutch Famine Birth Cohort have reported both in utero, intergenerational and transgenerational effects of famine to cardiovascular health [19, 27]. As previously mentioned, this cohort includes the children and grandchildren of women and men who conceived during the years of the Hongerwinter, a period of famine in the Netherlands caused by a German blockade during the Second World War. This design includes three different exposures: (1) early-life undernutrition, if the parents conceived just before this period; (2) in utero exposure to undernutrition, when pregnancy took place during the period; and (3) ancestral exposure to limited caloric intake, when parents conceived after the period of malnutrition. The health information on exposed individuals and their offspring was obtained via the historical records of the Dutch healthcare system, thus completing the information of this cohort [19, 27].

Interestingly, a similar cohort originated from the Great Chinese Famine (1959–1961) correlates pre-gestational undernourishment with increased risk of type 2 diabetes and hyperglycemia [28]. This experimental design draws associations of transgenerational effect of famine, contrary to the Dutch Cohort studies that evaluate in utero exposure, therefore an intergenerational exposure. However, the Chinese cohort study presents a pitfall due to the lack of medical records of the exposed individuals; therefore, it is not possible to evaluate the accuracy of selection criteria for exposed and non-exposed individuals. This aspect has been properly addressed in the earliest publication based on the Swedish Överkalix Cohort [3]. This comprising three birth cohorts 15 years apart from each other (1890, 1905 and 1920) was correlated with historical, agricultural, and socioeconomic records to estimate food availability during the slow growth period. Similarly to the Chinese Famine study, the Överkalix cohort study suggests that food availability has an impact in the onset of metabolic disease in the grandchildren via the paternal lineage. Yet, contrary to the Chinese cohort, overfeeding was the factor associated with higher cardiovascular risk in grandchildren [3].

The most cited publication in paternal epigenetic inheritance of metabolic traits is an ancestral exposure, associative non-multigenerational study describing the intergenerational effects of low-protein diet in hepatic gene expression of non-exposed offspring [17]. Here, male mice were fed low-protein diet or control diet for 6–9 weeks, before mating with control diet-fed females. The resulting male offspring were euthanized at 3 weeks of age and their tissues collected for evaluation of multiple metabolic parameters. The key finding is the differently DNA methylation rate in liver between the offspring of mice fed with low protein and the offspring of mice fed with standard chow, notably of an enhancer sequence associated with the Peroxisome proliferator-activated receptor α gene (*Pparα*). This is among the earliest experimental evidence of paternal inheritance of traits via an epigenetic mechanism (i.e., DNA methylation). Besides this highly cited publication by Carone et al. [17], only two other studies among selected publications describe exposure to low-protein diet [85, 86]. On contrary, HFD is among the most studied exposures. For instance, the first paper reporting epigenetic inheritance of acquired traits via sperm sncRNA, notably miRNA, adopted a model of ancestral exposure to HFD [37]. In this work, the authors have demonstrated that excess adiposity caused by HFD altered sperm miRNA content and germ cell methylation status that persisted up to 2 generations downstream the exposure. They further report an increase in adiposity of descendants of HFD-fed mice, compared to descendants of mice fed standard chow.

Among selected multigenerational exposure studies, only one reports a cumulative exposure effect mediated by sperm sncRNA [59]. The authors reported a progressive increase in fat mass and in the prevalence of related metabolic diseases over 5 generations of mice fed a Western-like diet. However, this cumulative effect seems to be reversed just within one generation of standard chow diet. Additionally, the authors have injected sperm sncRNAs isolated from sperm of mice exposed to western-like diet into naïve zygotes. As demonstrated in previous studies [43, 44], the injection of sncRNA isolated from sperm of exposed mice led to the expression of the exposure-like phenotype in the progeny. Interestingly, the phenotype was not transmitted to the offspring of the mice originated from manipulated zygotes [59]. These findings suggest that, despite mounting evidence of the sncRNA potential to transmit acquired traits when injected into the zygote, the amount of sncRNA carried by a single spermatozoon or post-fertilization events may be a challenge for this epigenetic inheritance mechanism in vivo.

Despite the growing interest on the role of microbiota in multiple systems, notably in the context of metabolic adaptation to dietary cues, just two publications on paternal epigenetic inheritance of metabolic traits were retrieved by our search strategy [87, 88]. In the study by Seth and colleagues [87], male mice were fed HFD or control diet for 16 weeks, then followed by a treatment with the probiotic *L. rhamnosus* for 12 weeks. Then, male mice were mated with unexposed females. The authors reported multiple differences in epigenetic marks (miRNA content, methylation, histone modifications) in liver and pancreas of mice treated with the probiotic comparing with untreated mice [87]. These changes were also observed between the offspring of treated versus untreated mice. The treatment with the probiotic has also promoted changes in sperm epigenetic signature. The other publication describing exposure to microbiota investigates the inheritance of immune response to infection, in this case, *C. albicans* [88]. For that, the researchers injected mice with *C. albicans* intravenously to induce a sublethal systemic infection. The offspring of male mice surviving the infection showed enhanced response to other systemic infections, comparing with the offspring of uninfected mice [88]. Systemic infection was also associated with changes in sperm miRNA content, suggesting that this is the vehicle for the epigenetic inheritance of enhanced response to infection [88].

### Exercise

Studies describing the effects of exercise in the paternal epigenetic inheritance of metabolic traits are mainly focus on “lifestyle intervention” to tackle excessive adiposity and the onset of metabolic syndrome. The earliest “lifestyle correction” study included in this systematic review describes an exercise program to revert the metabolic effects of excess adiposity on overweight, HFD-fed mice [89]. Mice were fed a control diet (6% fat) or high-fat diet (21% fat) for 9 weeks. HFD males were then allocated to diet and/or exercise interventions for nine more weeks. The exercise program improved metabolic and sperm parameters of the HFD-fed mice and their progeny, comparing to HFD-fed sedentary mice and their progeny. However, a recent study of diet correction reported opposite results, especially among animals fed with HFD [90]. In this study, a group of mice fed HFD from weaning reverted its diet to standard chow in early adulthood (60 days post-weaning age), whereas other groups were exclusively fed by HFD or standard chow. Contrary to the study of weight loss due to exercise, the authors have observed lower sperm motility, viability, and increased number of abnormal sperm even 140 days after reversion to standard chow. Diet reversion reduced adiposity and prevented metabolic syndrome-like phenotype, but an analysis of testicular metabolome revealed irreversible changes after HFD exposure during sexual maturation. Additionally, this testicular metabolic signature of HFD was transmitted to the offspring and grandoffspring, but it was only associated with a phenotype in the grandoffspring of HFD-exposed mice, who presented lower sperm counts than the grandoffspring of the chow-fed mice [90]. The findings of these publications highlight the challenges of lifestyle-based interventions and the complexity of epigenetic inheritance. The duration and timing of exposure and intervention significantly influence the efficacy of the intervention and the epigenetic fingerprint transmitted to the offspring.

### Psychological trauma

The second and the third most cited publications included in this systematic review are both intergeneration gamete exposure study describing the acquisition of traits via sncRNA [43, 44] (Additional file 1, table S1). For that, Gapp et al. [43] isolated sperm miRNAs from mice which suffered early life trauma (premature maternal separation) and have injected them into mouse zygotes generated from in vitro fertilization of gametes obtained from unexposed males and females. The offspring generated from the manipulated zygote developed the same stress-associated traits and metabolic alterations as the mice affected by early trauma, contrary to mice generated from unmanipulated zygotes. In another study associating psychological trauma with metabolic changes in unexposed progeny, researchers injected exogenous sperm sncRNA into zygotes to explore the impact of fear conditioning and training on two generations of mice [60]. The authors obtained sperm sncRNAs from two groups of mice: a group of fear-conditioned mice through continuous exposure to the scent of a predator, and another group of mice that received mild foot shocks while being exposed to the same scent. The isolated sperm sncRNA was then injected into zygotes that had not been previously exposed to these experiences to produce offspring. When offspring reached adulthood, they were exposed to the same scent as the sncRNA donor mice to assess their response to this simulated predator. Interestingly, the male offspring resulting from the manipulated zygotes did not exhibit an enhanced fear response after the conditioning program involving the scent and shocks. This suggests that the presence of the predatory scent across generations had inhibited the fear response to this stimulus in males, but not in females [60]. Together, these studies provide compelling evidence for the role of sncRNAs in mediating the transmission of psychological trauma-associated traits, further enriching our understanding of paternal epigenetic inheritance in the context of psychological stressors and metabolic outcomes.

### Exogenous sncRNAs and KO/KI’s

The studies using exogenous sncRNAs and genetically modified organisms with knock-out (KO) or knock-in (KI) of genes of interest provide valuable information on the mechanisms of epigenetic inheritance. More than half of the mechanistic studies included in this systematic review (19/35, 54.3%) adopted one of these strategies. Commonly, studies involving the injection of exogenous RNAs require previous exposure of donors to a stimulus of interest. This was the strategy adopted by Gapp and colleagues [43], in their work on the transmission of psychological trauma. In this study, the researchers exposed litters of generation F0 to early maternal separation (3 h/day in the first 3 weeks of life). These male mice were used to generate the F2 generation upon reaching adulthood, through mating with a non-exposed female. Sperm was collected from the adult mice of the F2 generation, and total RNA isolated. Total RNA pooled from five F2 mice of the ancestrally exposed lineage and from five F2 mice of the non-exposed mice was then injected into a zygote, fertilized by non-exposed mice. The authors observed that zygotes injected with RNA from mice ancestrally exposed to early maternal separation exhibited the same behavioral and metabolic phenotype as their grandfathers, the mice exposed to early maternal separation [43]. Chen et al. [44] demonstrated that sperm tRNA-derived small RNAs (tsRNA) were sufficient to transmit metabolic disorders onto the offspring. To do so, the researchers fed mice with HFD to induce a metabolic phenotype of pre-diabetes. Sperm was collected from mice and sperm RNA isolated. sncRNA sequences were then sorted to create subsets. Those sncRNA subsets were injected into mice zygotes obtained from in vitro fertilization of gametes from non-exposed males and females. The injection of the sncRNA portion enriched in tsRNAs, particularly 5’- transcription initiation RNAs (tiRNAs), led to the onset of the metabolic phenotype in the offspring, despite the progenitors not having it nor having been exposed to HFD. The authors further demonstrated that this epigenetic inheritance was unrelated to DNA methylation. A recent study used a genetically modified mouse overexpressing histone demethylase enzyme KDM1A to show that histone H3 lysine 4 methylation (H3K4me3) pattern is inherited by the offspring [91]. Male mice overexpressing KDM1A were fed HFD for 10–12 weeks to further increase total DNA and histone methylation, and then mated with chow-fed C57BL/6 NCrl female to generate the F1 offspring. These mice were later bred with another chow-fed C57BL/6 NCrl female to obtain the F2 generation. The authors have found that the overmethylation of histones, measured as H3K4me3 expression, is the main driver of epigenetic inheritance of the obesity-associated metabolic phenotype in unexposed progeny [91]. This paper mounts on knowledge from a previous paper, one of the top-10 cited papers in epigenetic inheritance of metabolic traits [92].

In conclusion, the utilization of exogenous sncRNAs and genetically modified organisms with KO or KI has proven to be instrumental in unraveling the intricate mechanisms of epigenetic inheritance. These approaches, accounting for over half of the mechanistic studies surveyed in this systematic review, have shed light on how paternal environmental exposures and genetic modifications can influence traits passed down through generations. Notably, the innovative strategies employed by researchers, such as injecting ancestrally influenced RNA into zygotes or exploring the role of specific sncRNA subsets, have yielded profound insights into transgenerational effects. Furthermore, the groundbreaking work on histone methylation patterns and their inheritance adds another layer to our understanding of epigenetic mechanisms. This body of research underscores the complex interplay between genetic and environmental factors in shaping the epigenetic landscape and, ultimately, the paternally inheritance of metabolic traits.

### Other exposures

The number of studies on the paternal epigenetic inheritance of metabolic traits is still small compared to other subjects, but the studies are diversifying. A significant number of publications (13.9%, 41/295) investigate the effects of exposures that do not fall within any of the main categories of exposures defined by us in this systematic review.

An example of this diversification comes from a human prospective multigenerational study that implemented a semi-mechanistic experiment [93]. In this work, the authors studied the inheritance of asymmetric methylation patterns in non-imprinted loci along a 4-generation family. They found that methylation patterns were kept in non-imprinted loci and that this methylation pattern was preserved between somatic and germ cells. This suggests that methylation patterns in humans are maintained even through the “epigenetic reset” of spermatogenesis, therefore suggesting a mechanism for epigenetic inheritance via the male gamete in a human family [93]. Another interesting and recent paper describes the intergenerational effects of exposure to radiation in a Russian cohort [94]. This retrospective (historical) study based on the Ozyorsk Children’s Health Registry associated the prevalence of endocrine and metabolic pathology in children born and living in Ozyorsk between 1949 and 1973, and preconcepcional exposure of their parents to gamma radiation at the Mayak Production Association, the first atomic industry facility in Russia. The researchers found that children of parents working at this nuclear industry had higher prevalence of thyroid diseases, including cancer, comparing to the offspring of low exposed parents—inhabitants of Ozyorsk but not working at the Mayak Production Association [94].

Parental age is rising worldwide, which raises concerns about the effects of advanced paternal age in the health of the offspring. In fact, a comprehensive review on the effects of aging in the male gamete and in the offspring was recently published by *Human Reproduction Update* [95]. A prospective study published in 2007 already associated smoking habits and age with the prevalence of type 1 diabetes in the offspring, in two British cohorts [25]. This study contained more than 11,000 valid subjects, but the overall prevalence of type 1 diabetes was below 1% [25]. The authors did not find any correlation between paternal (and maternal) age and type 1 diabetes prevalence, whereas they found paternal smoking habits as a protective factor against the onset of the disease in the offspring [25]. However, as discussed by the authors, the low prevalence of type 1 diabetes greatly reduced the power of the analysis, limiting the validity of the conclusions [25]. Another study of the effects of advancing paternal age and its effects on the health of the offspring compared sperm methylation of men with young paternal grandfathers and men with old paternal grandfathers [30]. This study recruited two small groups of patients attending a fertility clinic in US accordingly divided into two groups: old paternal grandfather age (> 40 years old) and young paternal grandfather age (< 25 years old). The age of the grandfather (F0) refers to the age he fathered the father (F1) of the patient (F2). The authors have not found any differences in sperm methylation between the two groups, but the sample size is too small, and the population possibly skewed due to possible fertility issues [30]. Advanced paternal age studies were also conducted in other mammalians. A recent study by Mao et al. [96] showed that the offspring of 21-month-old mice manifests several metabolic alterations, such as impaired glucose and lipid homeostasis, comparing to the offspring of 3-month-old mice [96]. The authors further report changes in liver and sperm gene expression annotated to lipid metabolism, thermogenesis, cholesterol metabolism, type II diabetes mellitus and endocrine resistance [96].

In summary, while the body of research on paternal epigenetic inheritance of metabolic traits remains relatively small compared to other subjects, it is diversifying and uncovering intriguing insights. A notable example is a human multigenerational study that delves into the inheritance of asymmetric methylation patterns in non-imprinted loci, revealing that such methylation patterns persist across generations, even through the process of spermatogenesis. Furthermore, as the global trend of increasing parental age continues, studies examining the effects of advanced paternal age on offspring health have gained prominence. Research in rodents has provided compelling evidence of metabolic alterations in offspring associated with advanced paternal age, underscoring the importance of understanding the impact of age on paternal contributions to metabolic traits in mammals. This evolving body of work promises to enhance our comprehension of epigenetic inheritance’s intricacies and its implications for health across generations.

## Vehicle/mechanisms of inheritance

An important element of epigenetic inheritance is the vehicle of this inheritance, i.e., how changes caused by exposures can be inherited by unexposed progeny. Interestingly, less than a third of all the publications in paternal epigenetic inheritance of metabolic traits (29.5%, 87/295) report changes in the vehicle of epigenetic inheritance (sperm). These changes are reported as outcomes of exposure to the stimulus in study. The most reported changes are related to sperm DNA methylation (58.6%, 51/87). Sperm miRNA and tsRNA content follow the most measured changes (20.7%, 18/87 and 14.9%, 13/87 respectively). A series of studies evaluating the transgenerational effects of embryo exposure to multiple environmental toxicants reported changes in sncRNA content, methylation rate, methylated regions, histone retention, and histone methylation in sperm of grand-grandoffspring of exposed females comparing to the grand-grandoffspring of non-exposed females [47, 48, 50, 54]. In another series of publications, the HFD-fed mice displayed changes in sperm sncRNA content, compared to non-exposed mice [97]. The offspring and grandoffspring of the exposed mice also displayed changes in the sperm sncRNA content, comparing to the descendants of non-exposed mice [97]. Interestingly, the differently expressed sperm sncRNAs found in each generation was not reflected in other generations, raising the question whether these changes are directly responsible for a phenotype and for the inheritance of the traits of ancestral exposure to HFD. Similarly, Chen et al. found changes in sperm tsRNA content in mice fed a HFD [44]. Starting from this observation, the authors hypothesize that this sperm sncRNA signature was involved in the transmission of the HFD-associated phenotype to unexposed progeny. To test this hypothesis, sperm tsRNAs from chow-fed and HFD-fed mice were extracted and injected in naïve zygotes. Psychological stress was also shown to cause changes in sperm sncRNA content, notably in miRNAs. Gapp and colleagues found differently expressed sperm miRNAs between adult mice which suffered premature maternal separation, comparing to non-exposed mice [43]. Then, sperm RNA content of traumatized mice was isolated and injected in naïve mouse embryos to assess if trauma-associated behaviors were observed in mice generated from manipulated zygotes, comparing to mice generated from unmanipulated zygotes [43].

## Measured outcomes

### Phenotype

The majority of studies encompassed within this systematic review primarily assess phenotypic variations among exposed and non-exposed individuals and their subsequent generations. Notably, we include sperm parameters as a facet of phenotype measurement, recognizing that they are reflective outcomes influenced by a multitude of factors encompassing metabolism, endocrinology, and reproduction. It is worth highlighting that the literature on paternal inheritance of metabolic traits presents an array of conflicting findings regarding sperm parameters and male fertility in the offspring. This disparity persists even when considering studies that delve into similar exposures, emphasizing the complexity and variability within this field of research. For instance, streptozotocin-induced diabetes was reported to decrease sperm concentration up to two generations after exposure (offspring and grandoffspring) [98]. However, diet-induced diabetes did not decrease sperm concentration in the offspring of exposed mice comparing to the offspring of chow-fed mice [90]. However, the grandoffspring of HFD-fed mice and had lower sperm counts than the grandoffspring of chow-fed mice [90]. In both studies, mice exposed to metabolic syndrome did not suffer any changes in sperm counts, but sperm morphology and motility were altered [90, 98]. The intergenerational inheritance of acquired phenotypes was also reported in the offspring of rats exposed to xenoestrogens during embryo development [58]. In this study, the authors observed an increase in the number of apoptotic and binuclear hepatocytes, and accumulation of γ-glycogen in the liver of the offspring of mice exposed to xenoestrogens in utero, comparing to the offspring of non-exposed mice [58].

### Behavioral outcomes

Behavioral outcomes are usually described in publications where the initial exposure was related to psychological trauma, such as stress or fear. To measure these effects as outcomes, researchers employ a plethora of tests to assess social behavior, spatial recognition, addiction, and overall brain functioning. The first paper implementing a strategy of exposure, isolation of sperm RNA from exposed individuals, and injection of isolated RNAs into naïve zygotes aimed at understanding the mechanisms of intergenerational inheritance of psychological trauma [43]. In this study, sperm miRNAs of mice which suffered premature maternal separation were injected in zygotes resulting from gametes of non-traumatized mice. Notably, adult mice resulting from the manipulated zygotes showed the same stressed, anxiety-like behavior as the adult miRNA donors [43]. This behavior was not observed in adult mice generated from unmanipulated zygotes, thus suggesting that paternal miRNA content conveys the information for the development of the psychological phenotypes in mice. In another study, researchers suggested that a fear conditioning program could be transmitted epigenetically, thus enhancing the response to a threat [60]. To do so, mice were subjected to fear conditioning by chronic exposure to the odor of a natural predator or paired exposed to odor and mild electric shock. Sperm RNAs of these mice were collected and injected into naïve zygotes. Later, mice originated from the manipulated zygotes were exposed to the same fear conditioning program to measure their response to the stimuli [60]. The authors found that adult mice originating from manipulated embryos had increased sensitivity fear, but male mice do not consolidate the response to fear paired with pain, contrary to females [60]. Another interesting behavioral outcome is “drug self-administration,” a common measure in studies about addiction. A rodent study of embryo/gamete exposure to cocaine shows that the offspring of male mice exposed to cocaine in adult (and during mating) is associated with decreased cocaine self-administration in the offspring [99]. Curiously, this trend is also observed in similar studies involving consumable drugs [84, 100] but it is opposite in studies of self-administration of HFD [101].

In conclusion, the examination of behavioral outcomes in the context of paternal inheritance unveils a multifaceted landscape of findings. Researchers employ a diverse array of tests to gauge social behavior, spatial recognition, addiction propensity, and overall brain functioning as endpoints. These remarkable discoveries emphasize the role of paternal factors in shaping behavioral traits across generations. Furthermore, investigations into the epigenetic transmission of fear conditioning have elucidated gender-specific responses, underscoring the intricate nature of these inheritable traits. Notably, studies exploring drug self-administration have revealed intriguing trends, including decreased cocaine self-administration in offspring exposed to cocaine via their male parent, demonstrating the far-reaching implications of paternal influences on addiction-related behaviors. However, the complexity of these outcomes is further underscored by differing trends observed in studies involving consumable drugs versus high-fat diets, adding to the rich tapestry of knowledge on behavioral outcomes in the context of paternal epigenetic inheritance.

### Metabolic and endocrine changes

The focus of this systematic review revolves around the transmission of metabolic traits, influenced by various exposures, to unexposed offspring. Nevertheless, not all the publications encompassed within this review exclusively document metabolic alterations as the primary outcome. It is important to note that within our criteria, changes in BMI, body weight, and fat mass were not classified as metabolic changes. Instead, we categorize the emergence of metabolic syndrome, glucose intolerance, and insulin resistance as metabolic outcomes, as they provide valuable insights into overall metabolic function. Additionally, we have included assessments of gene and protein expression within this outcome category, as they serve as indicators of physiological processes within specific tissues and organs. This comprehensive approach ensures a thorough examination of the multifaceted aspects of metabolic inheritance across the reviewed studies. Studies focused on the effects of diet in unexposed progeny often report measures of metabolic function in the progeny. However, the metabolic outcomes of epigenetic inheritance of exposure to diets vary according to the severity and duration of the exposure, and the age or life stage of the subjects. For instance, regarding ancestral exposure to HFD, Crisóstomo et al. [90] did not observe any phenotype related to metabolic syndrome in the offspring and grandoffspring of mice fed a HFD, comparing to the offspring and grandoffspring of mice fed with control diet [90]. This group has also measured endocrine outcomes, namely serum concentrations of insulin, testosterone, and estradiol, but no endocrine changes were found. However, there were differences in the relative abundance of several testicular metabolites in every generation, when comparing the offspring and grandoffspring of HFD-fed mice against the offspring and grandoffspring of chow-fed mice [90]. The cumulative effects of HFD were also studied, using a Western diet exposure rodent model [59]. As in many other multigenerational exposure studies, the authors tested whether the exposure to a stimulus (in this case, HFD) over multiple generations could lead to more severe phenotype in unexposed offspring. Besides that, the authors suggested that longer exposure (i.e., exposure over more generations) could extend the HFD-related phenotype to more non-exposed generations. Interestingly, no differences in glucose homeostasis parameters were found between mice exposed to HFD for one generation or multiple generations [59]. Glucose homeostasis was impaired in HFD-exposed mice compared to chow-fed mice. Moreover, the offspring of HFD-fed mice retained the metabolic syndrome-like phenotype comparing to the offspring of chow-fed mice [59], similarly to other reports [90]. On the other hand, the metabolic phenotype was not inherited transgenerationally after just one generation of chow diet, even after several generations of HFD exposure. Hence, the authors suggest that multigenerational exposure to HFD does not yield cumulative effects in severity and extension of the HFD-associated metabolic phenotype in unexposed offspring [59]. This diversity in findings underscores the complexity of the field and highlights the need for further investigation to elucidate the mechanisms underlying metabolic inheritance across generations.

### Non-coding RNAs

**Fig. 4:**
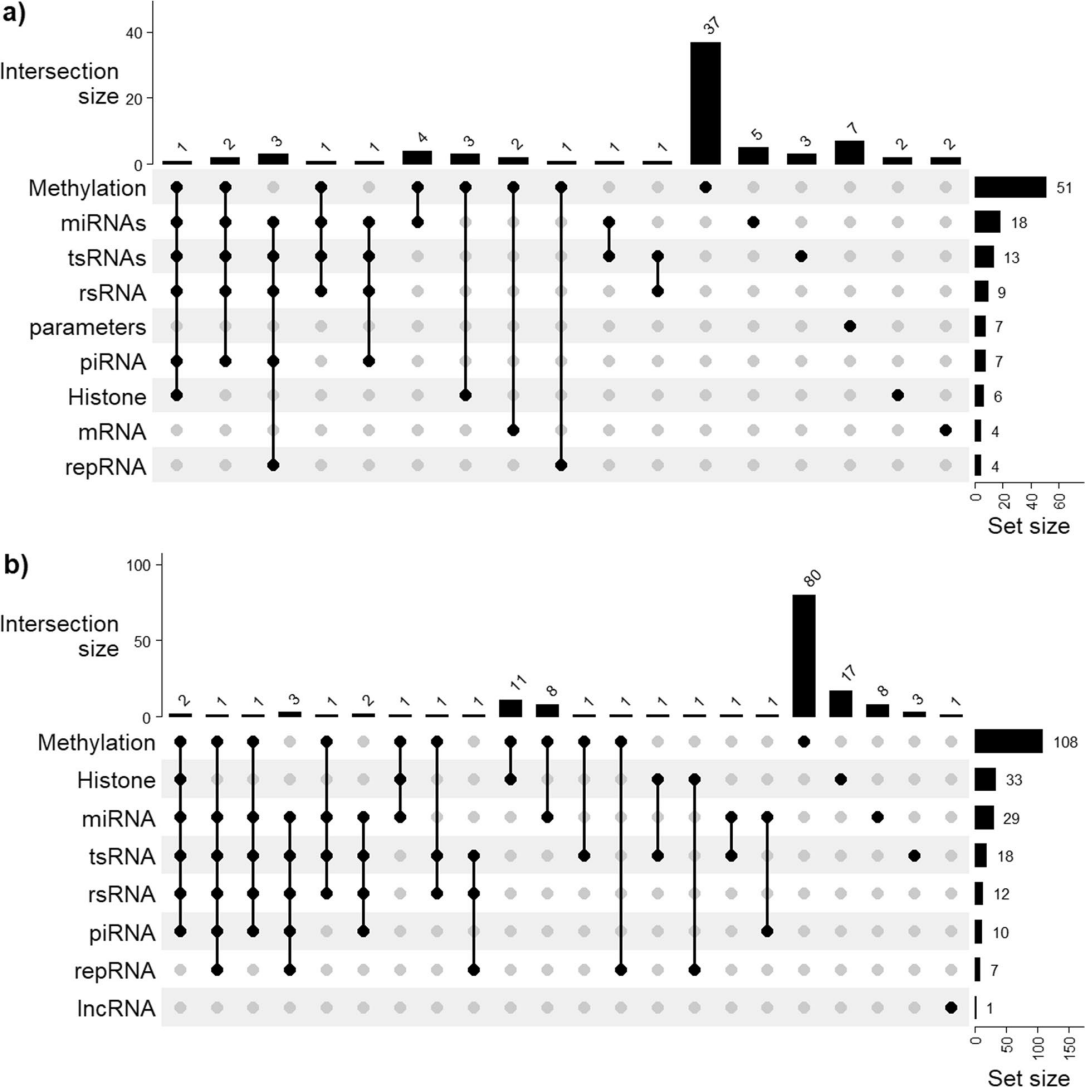
Detailed measures of change in **a** vehicle of epigenetic change (sperm) and in **b** outcomes of epigenetic inheritance mechanisms in the offspring. DNA methylation and mechanisms leading to DNA methylation are the most commonly measured change in both the vehicle and the outcome of studies on the paternal epigenetic inheritance of metabolic traits

Non-coding RNAs are the second most reported epigenetic outcome among the publications considered for this systematic review (10.5%, 31/295) (Fig. 4b). The earliest evidence of the role of sperm sncRNA in the paternal epigenetic inheritance of metabolic traits is found in a study by Fullston et al. [37]. In this study, the authors fed mice a HFD or chow, and assessed sperm and germ cell sncRNA content and methylation, from the exposed generation up to their non-exposed grandoffspring [37]. The authors found several differentially expressed sperm miRNAs down the HFD lineage, comparing to the offspring and grandoffspring of chow-fed mice. Gapp et al. [43] have also found a sperm miRNA signature of early trauma, whereas Chen et al. [44] found a sperm tsRNA signature of HFD. In both publications, the injection of the altered sncRNA fraction into naïve zygotes resulted in the resume of the phenotype observed in the exposed mice [43, 44]. But other sncRNA fraction have the potential to transmit epigenetic information of metabolic traits via the male gamete, as suggested by Crisóstomo and colleagues [97]. This study includes small RNA sequencing data obtained from an ancestral HFD exposure study [90, 102] and shows that ancestral HFD exposure causes a dynamic sperm sncRNA signature along two non-exposed generations of mice [97]. The authors report mostly changes in sperm tsRNA content in the exposed generation, but they also report that sperm piRNA and repRNA content is the main source of differences in the offspring of exposed versus non-exposed mice, whereas changes in miRNAs persist in the grandoffspring of exposed mice comparing to the grandoffspring of chow-fed mice [97]. Other authors have also reported changes in sperm rRNA-derived small RNAs (rsRNAs) in response to diet in both mouse [103] and human [104]. Moreover, recently developed advanced small RNA sequencing protocol by overcoming RNA modifications has revealed that rsRNAs are in fact most abundant in mature sperm [105]. The complexity of sperm small RNAs and modifications has been referred to as “sperm RNA code” which are essential in transmitting paternal metabolic phenotypes [106].

Our bibliographical search has just retrieved one publication reporting epigenetic inheritance of changes in the expression of long non-coding RNAs (lncRNAs) [107]. This study, conducted in mice, investigated the effects of HFD in mRNA and lncRNA expression in testis of exposed subjects and their progeny. The authors found multiple differently expressed RNAs related to endocrine and metabolic pathways, notably, the GnRH and Hippo signaling pathways [107]. Overall, the wealth of findings regarding ncRNAs underscores their pivotal role in mediating paternal epigenetic inheritance and highlights the intricate “sperm RNA code” as a key player in transmitting metabolic traits across generations.

### Methylation

DNA methylation, or another metric related to DNA methylation such as DNA methylase activity, is the most commonly epigenetic outcome measured in our selected studies (108/295, 36.6%). More than a third of the papers reporting epigenetic changes (80/218, 36.7%) exclusively measure changes in methylome of target tissues (Fig. 4b). The highly cited paper by Carone et al. [17] perfectly illustrates this trend. In this study, the authors observed a different DNA methylation rate in livers of the offspring of mice fed with low protein and the offspring of mice fed with standard chow. Notably, the enhancer sequence associated with the *Pparα* was differently methylated between the compared groups. This is one of the earliest experimental evidence of paternal inheritance of traits via an epigenetic mechanism (i.e., DNA methylation). Previously, this phenomenon was described in a paper by Rakyan and colleagues [18], who exploited the phenotypic variability associated with the axin-fused rodent allele to first describe the inheritance of methylation patterns in the male germ line. This intergenerational mechanistic study investigated the methylation status of the axin-fused allele in mice displaying the associated phenotype—a kinked tail—and mice not displaying the phenotype. The authors have identified that the methylation of a retrotransposon within the allele was responsible by the expression of the phenotype. The authors have then bred the animals and have found that the methylation status was preserved both via the maternal and the paternal gamete, providing evidence that methylation patterns in some *loci* are not erased during gametogenesis. The mechanisms of epigenetic inheritance via methylation patterns were further described by Grandjean et al. [108]. In their paper, the authors have knocked-out genes encoding DNA methyltransferases (*Dmnt1*, *Dmnt2*, and *Dmnt3a*) in mice, therefore impairing their capacity to methylate DNA regions. In mammalians, Dmnt1 is responsible for the methylation of CpG islands [109], while other DNA methyltransferases are associated with methylation of non-CpG motifs [110]. Surprisingly, the authors found that the Dnmt1 knock-out did not interfere with the inheritance of CpG methylation of the *Rxrα* locus, but it abrogated non-CpG island methylation in the progeny. In contrast, the offspring of *Dmnt2* and *Dmnt3a* null mice inherited the *Rxrα* locus methylation pattern of the male founder. These data suggest that some methylated *loci* are not erased during spermatogenesis. Moreover, preserved methylated CpG may be recognized by DMNT1 to reestablish the methylation pattern of the locus, even beyond CpG sequences. The mechanisms of DNA methylation via DMNT1 and DMNT2 enzymes was further studied in two other paternal inheritance studies [103, 111], and gene expression of DNA methyltransferases has been recently adopted as an exposure outcome metric in paternal inheritance experiments [112–115].

An interesting mechanistic work on the importance of DNA methylation in epigenetic inheritance was conducted by fertilizing murine oocytes via round spermatid injection (ROSI) [116]. In this study, the authors suggest that bypassing the gamete epigenetic reprograming checks leads to an increase in embryo malformation rate due to abnormal DNA methylation. The authors observed that the methylation mark H3K27me3 persists in ROSI-fertilized embryos, while this mark decreased in normal spermatozoa. H3K27me3 is reduced during spermiogenesis due to histone turnover and replacement by protamines. ROSI-fertilized embryos keeping elevated levels of H3K27me3 showed changes in chromatin accessibility comparing to normal embryos, therefore impairing gene transcription [116]. Recently, another group further elucidated the preservation of methylated loci through spermatogenesis and embryo development [117]. Collectively, these findings underscore the intricate interplay of DNA methylation in the inheritance of metabolic traits via paternal epigenetic mechanisms.

### Histones and chromatin

Histone modifications, histone turnover and substitution by protamines, and global chromatin condensation are another mechanism of epigenetic inheritance frequently reported as an outcome of ancestral exposures. A recent study by Pepin and colleagues [91] employed genetically modified mice overexpressing the histone demethylase KDM1A and ancestral exposure to HFD to study the effects of excessive DNA and histone demethylation in the metabolic health of unexposed progeny, up to the second non-exposed generation. The authors found that the joint action of the environmental exposure (HFD) and KDM1A overexpression led to excessive removal of histone H3 lysine 4 methylation (H3K4me3) in sperm, embryo, and placenta, particularly in regions related to metabolic, inflammatory, and developmental processes [91]. The authors further associate the H3K4me3 histone modification with chromatin accessibility. These findings resonate with the conclusions of another recent paper [118]. In this work, the authors investigated the same histone modification, H3K4me3, but using a model of folate-deficient diet. This nutritional deficit changes methyl donor availability, thus promoting alterations in the sperm and embryo epigenome. The authors observed that a proportion of the changes in total H3K4me3 are retained in sperm and in the pre-implantation embryo, being associated with impaired embryonic gene expression. Similarly to Pepin and colleagues [91], a mice model overexpressing H3K4me2/3 marks was used to confirm the cumulative effects of “basal” histone methylation processes and HFD. Based on that, the authors demonstrate that H3K4me3 alterations are retained and linked to dysregulated gene expression in sperm and in embryo and that those changes are associated with metabolic phenotypes via alterations in chromatin condensation state [118].

Another study reported defects in histone modification by protamines using a mixed exposure mice model [98]. In this model, type 1 diabetes was induced by streptozotocin, and sperm and testis histones of the diabetic mice, their offspring and grandoffspring were characterized. The authors found differences in the protamine 1 to protamine 2 ratio in the sperm of diabetic mice comparing to healthy mice, which were later associated to decreased sperm quality and testis transcriptome in the offspring and grandoffspring [98]. A similar effect was found in the offspring of undernourished pregnant mice [119], although this study refers exclusively to maternal exposure.

### Other outcomes and sex-specific differences

Besides the outcomes already discussed in this systematic review, there are other less represented outcomes in our final selection of publications on the paternal epigenetic inheritance of metabolic traits. An interesting and increasingly popular outcome is gut microbiota (2.4%, 7/295). Most of the studies reporting gut microbiota as an outcome refer to diet-related exposure [113, 120–122] but, interestingly, it is often associated with models of psychological trauma [123, 124]. However, one of the studies associating psychological stress and microbiota does not quantify changes in gut microbiota—the authors report changes in urine metabolites between the offspring of expose and non-exposed father that are associated with microbial host interactions [124]. Despite that, the authors apply two complex epigenetic inheritance models, also including maternal inheritance, having found similar changes related to microbial host interaction in both models of exposure to psychological stress [124]. Another interesting paper on microbiota investigates the role of microbial choline consumption in the gut to epigenetic and metabolic homeostasis using a rodent model and transplant of microbiota unable to metabolize choline [125].

**Fig. 5:**
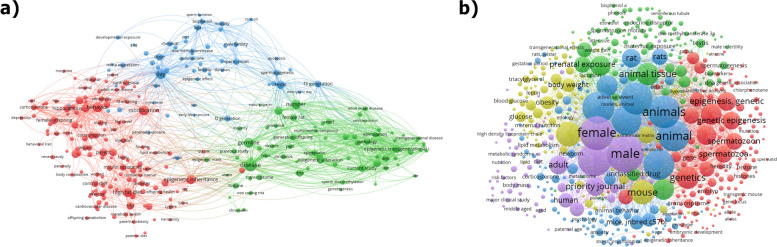
Semantic analysis of selected publications. **a** Semantic analysis of the abstract content, author keywords and index keywords, based on binary term co-occurrence. Three clusters of terms highly associated form – Red: metabolism-related terms; Blue: Vehicle-related terms; Green – Terms related to the characteristics of the study. **b** Semantic analysis based on term co-occurrence of terms in author-defined keywords. Using author-defined keywords, the terms cluster even more closely together. The terms are grouped as five clusters: Blue – Subject and psycho-behavioral terms; Red – Terms related with (epi)genetics and epigenetic transmission; Purple – Subject and metabolic-related terms; Yellow – Glucose, diabetes and obesity; Green – Terms related to environmental exposure to toxicants

Multiple studies report phenotypic differences between progeny of opposite sex. Although this review exclusively includes publications on paternal epigenetic inheritance, some publications report outcomes in both male and female progeny. Indeed, one of the most frequent terms in the abstracts of the selected publications is “female” (Fig. 5). This is the case of multiple papers studying the transgenerational effects of ancestral exposure to environmental toxicants authored by M.K. Skinner [49, 51, 52, 126]. Globally, female rats originated from the exposed male lineage are not as susceptive to pathology at any tissue than their male littermates [49, 51]. There are also conflicting data on these effects: ancestral exposure to atrazine led to lower body weight in females than in males of the same litter [51], whereas exposure to vinclozolin led to increased prevalence of obesity among females comparing to their littermates [49]. In both studies, these results refer to the same generation F3, grandoffspring of the exposed male F1, which was exposed during embryo development. Sex-based differences are also found in human studies. Two of the publications pertaining the Dutch Famine Cohort reported that the prevalence of cardiovascular and metabolic diseases among the sons of men exposed to famine in utero is not different from the prevalence among sons of unexposed men [31, 38]. Yet, the daughters of exposed men are more susceptible to cardiovascular diseases than the daughters of non-exposed men [31, 38].

Aoued et al. demonstrated that mice of different genders respond differently to intergenerational stress [60]. The researchers adopted a chronic stress model by exposing male mice to the odor of predator followed by zygote injection of sperm-borne sncRNAs purified from the exposed mice. The resulting offspring was subjected to the same chronic stress and a stress conditioning program where the predator odor was paired with mild electric shocks. The males resulting from manipulated zygotes did not show enhanced consolidation fear response after the conditioning program. Interestingly, females consolidated their fear response after the program. These results suggest that the presence of the predatory odor over generations inhibited the fear response to this stimulus in males but not females [60].

## Multiomics integration of published data obtained from the same animal model

### Redundant publications in epigenetic inheritance: are we missing the “full picture”?

The number of publications on paternal epigenetic inheritance is limited as demonstrated in this review. The complexity of the experimental designs and the large datasets produced by the monitoring of the model and the different experiments are challenging to interpret and publish. To overcome the content limits of scientific journals and to simplify the research, often authors opt to publish research in paternal epigenetic inheritance as a series of papers addressing a specific aspect of the model. Within the publications shortlisted in this systematic review, this practice is easily spotted, reducing the de facto number of publications in the subject. This practice might be considered close to “salami publishing” [127], although the hypothesis and the methods are not shared across the several publications. Still, the characteristics of transgenerational studies and publishing policies justify the data slicing in this context. However, with the development of complex multivariate statistical methods the publication of these data across different publications hinders conclusions that could be drawn if it would have been integrated using multivariate methods.

A meta-analysis based upon the publications meeting our inclusion criteria would lack power due to its limited size. Nevertheless, it is possible to combine, integrate, and extract information from publications referring to the same set of samples and individuals. Future meta-analyses on the subject must acknowledge and apply strategies to tackle the bias created by closely related publications. Hereby, we suggest a strategy based on multiomics integration applied to a set of published papers of our authorship [90, 97, 102, 128, 129]. There are, however, multiple examples of publications pertaining to the same model among the selected publications. Examples include a series of papers by M.K. Skinner group on glyphosate exposure [130, 131] and DDT/Vinclozolin exposure [41, 132], and analysis/reanalysis pair of papers by seemingly independent research groups [133, 134]. However, it is not easy to find all the papers pertaining to the same animal model. In those examples, the animal license number provided a reliable element to find other closely related publications.

### Model characterization and data selection

We have designed a mouse model of paternal ancestral exposure to HFD consisting in 3 generations. Summarily, we have assigned 36 male mice to 3 different diet regimens, after weaning (exposed F0 Generation): standard chow for 200 days (CTRL), high-fat diet for 200 days (HFD) and HFD for 60 days, then replaced by standard chow (HFDt). At 120 days post-weaning age, males were mated with same age, normoponderal, non-exposed females to generate the F1 Generation. The process was repeated with F1 males to generate the F2 Generation. Both F1 and F2 mice were fed standard chow for 200 days from weaning. More details of the model are available in the publications included in this systematic review [90, 97]. Three other publications included in this example were not retrieved by the bibliographical search strategy defined for this systematic review. Two of them exclusively describe the effects of HFD and HFDt in the exposed mice [128, 129], whereas the other publication does not include any of the terms considered in the bibliographical search [102].

We have exclusively selected publicly available data from our publications [90, 97, 102, 128, 129] for multiomics integration, although we have used raw data to apply a different normalization method prior to integration, as described in the “Methods” section. However, multiomics integration could be performed relying solely in the means, standard deviations, and number of biological replicates reported in the publications. With those statistical descriptors, it is easy to generate a dataset with similar characteristics using R, SPSS, or other statistical software. In this example, we have selected data from testicular metabolites obtained by ^1^H-NMR, testicular fatty acids obtained from GC–MS, and various fractions of sperm sncRNAs obtained from Next-Generation RNA-seq. The multivariate analysis of selected datasets was performed using sPLS-DA. This method is a supervised tool based on machine learning to highlight sources of variability between different groups [135]. The “sparse” component improves the discriminant power by reducing the weight of variables with low influence on the model [136]. A drawback of the method is the need of equal number of samples across blocks (datasets). In this example, we had to exclude samples with metabolomic and lipidomic data to meet the biological replicates of sperm sncRNA sequencing (*n* = 3 per group in Generation F0 and F2, *n* = 2 per group in Generation F1).

### Multiomics integration highlights sources of variation between mice exposed to different diets and their progeny

**Fig. 6:**
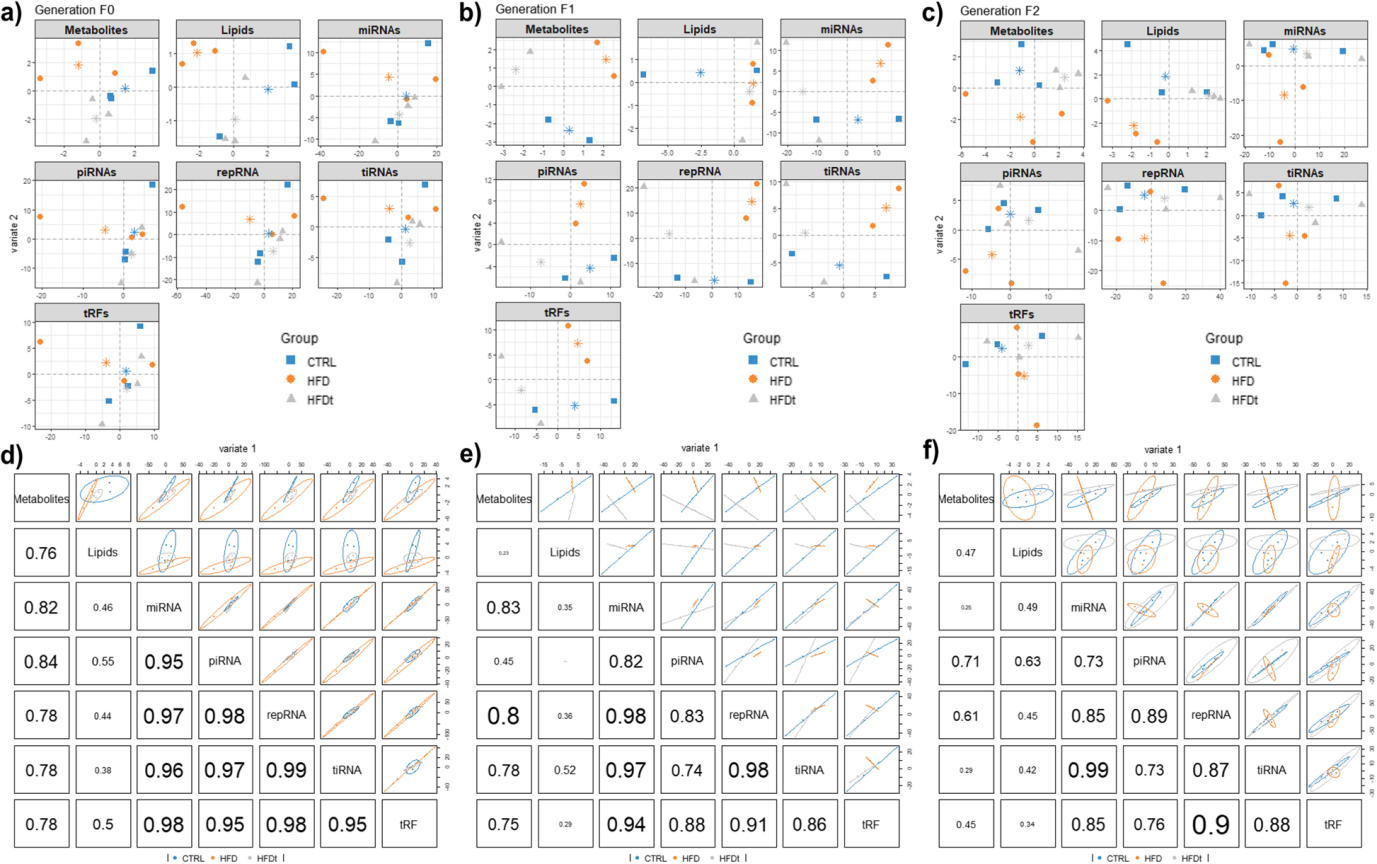
Multiomics integration of data from different papers based on the same animal model using sparse Projection of Latent Structures – Discriminant Analysis (sPLS-DA). Individual block plots of samples in the two-dimensional space spawned by the 2 first Latent Structures of **a** Generation F0, **b** Generation F1, and **c** Generation F2. sPLS-DA requires an equal number of samples in each block (dataset), thus the most representative samples according to metabolomic and lipidomic profile were selected using 2-omics PLS. The closest samples to the centroid of the group were selected to match the number of sncRNA-sequenced samples. Due to the integration of more datasets, the clustering of samples belonging to the same group (CTRL, HFD, and HFDt) is enhanced, particularly in the F1 generation. The block correlation plots of **d** Generation F0, **e** Generation F1, and **f** Generation F2 highlight each datasets share most similarities among each other, based on the first latent structure. Low correlation values suggest sources of differences among samples from different groups. Number of replicates: F0_CTRL = 3, F0_HFD = 3, F0_HFDt = 3; F1_CTRL = 2, F1_HFD = 2, F1_HFDt = 2; F2_CTRL = 3, F2_HFD = 3, F2_HFDt = 3

We have previously demonstrated that exposure to HFD, even after reversion to standard chow, is associated with a testicular metabolic and lipidomic signature that is transgenerationally inherited via the paternal lineage up to the grandoffspring [90, 102, 128, 129]. We have then reported that sperm sncRNA content was also affected by the exposure to HFD and HFDt, but it was not possible to relate differently expressed sperm sncRNA with the testicular metabolic phenotype [97]. Using multiomics integration to combine the datasets obtained using the different techniques and tissues (testis and sperm), it was possible to obtain a wider view over testis-spermatozoa interactions that support the phenotypic observations previously reported (Fig. 6). The projection of the two first latent structures obtained for each dataset in Generation F0 (Fig. 6a) is coherent to our previous reports, highlighting the differences in testicular metabolome and lipidome, but few differences in sperm sncRNA content. This is further evidenced by the block correlation plot of the first latent structure (Fig. 6d), where all sncRNA fractions achieve very high correlation levels, therefore not contributing for group separation. Opposing, lipids have low correlation coefficients with all other datasets, thus is the major source of difference between groups. The most interesting finding in this integration is the clear separation of samples belonging to different groups in generation F1 (Fig. 6b, e). Previously, we have found an evident overlap between the testicular metabolomic and lipidomic signature of the offspring of HFD, HFDt, and CTRL mice. Despite the large number of differently expressed sperm sncRNAs found between the offspring of HFD and HFDt, we have not found a clear separation between groups either. But when integrating together, the samples of the offspring of HFD are always plotted together in a cluster well-separated from the HFDt and CTRL clusters (Fig. 6b). Analyzing the correlation plots (Fig. 6e), it is further evident that HFDt samples do not correlate well with CTRL, despite their partial overlap. Regarding Generation F2, the multiomics analysis corroborates our previous findings, but highlights a total separation between groups in terms of testicular metabolite content, whereas testicular fatty acid content is more similar between grandoffspring of CTRL and HFDt. Interestingly, sperm miRNA content is similar in CTRL and HFDt groups, despite us having only identified differently expressed miRNAs between those groups in Generation F2 [97]. The block correlation plot (Fig. 6f) suggests that testicular metabolome and lipidome are the major sources of variation between grandoffspring of CTRL, HFD, and HFDt.

The multiomics integration of data from five different publications obtained from the same animal model has provided a broader view of the interactions between metabolome, lipidome, and sncRNAs. We have reported that exposure to HFD and HFDt affects testicular metabolism and consequently sperm parameters and that those traits were partly inherited by the grandoffspring [90, 102]. However, our findings do not follow the expected directionality of the HFD-related phenotype in testis. The weak phenotype expressed by the F1 offspring and the unclear separation of samples by groups according to their sperm sncRNA content challenged the hypothesis of epigenetic inheritance of the metabolic phenotype via the male gamete. Observing the “full picture” by integrating all datasets, the results suggest that a silent epigenetic fingerprint is inherited by the F1 generation and it is reflected in sperm sncRNA content. This complex epigenetic signature will contribute for the reappearance of the phenotype in the following F2 generation, but the mechanisms responsible for this epigenetic regulation remain not fully understood. This method has some limitations, especially the small number of samples that could be selected to perform the multivariate profiling. Nevertheless, multiomics integration provides a more reliable method to combine multiple papers and reconstruct the “full picture” of a transgenerational study.

## Conclusions

Phenotype results from a complex interaction of genome, epigenome, and environment, as postulated by Conrad Hal Waddington even before the “DNA Era.” There is now mounting evidence that acquired traits can be inherited or influence traits for several generations downstream an environmental exposure. However, the concept of epigenetic inheritance still faces challenges among the scientific community. The initial focus on intergenerational inheritance studies targeted gestation, due to the direct influence over the environment of the developing embryo. Due to this assumption, the number of publications in epigenetic inheritance is still biased towards the study of the matrilinear effects. However, it is now accepted that the male gamete can also transmit key epigenetic markers with the potential to transmit traits acquired (or modulated) by the environment.

After these findings, the number of publications in paternal epigenetic inheritance has expanded quickly, particularly in the context of the metabolic outcomes in the offspring. There are, however, other challenges to be addressed. Despite the variety of experimental designs, exposures, and outcomes, present literature is still dominated by a limited number of research groups which have found in the epigenetic inheritance their research niche. The collaboration between the most active groups in paternal epigenetic inheritance research while may expand the scope of their research, might also increase the risks of “scientific inbreeding”. Current literature exhibits already a bias towards studies of exposure to environmental toxicants and high-fat diets, and towards the outcomes in DNA methylation of onset of the disease. This implicates that there is insufficient evidence about other environmental exposures, such as psychological trauma, undernutrition and exercise, and about other outcomes, such as sperm sncRNA and long non-coding RNA content, DNA accessibility, and chromatin state. This skewness may be aggravated if the few research groups working on the subject start working in the same projects. Another matter of caution is the insufficient number of mechanistic studies compared to the number of ecological studies, regardless of the experimental design adopted. New experimental models could also be implemented, especially concerning the time-lapse of epigenetic modifications during spermatogenesis, fertilization and embryogenesis, to study the impact of the epigenome in the biological processes. Besides these issues, as demonstrated by the multiomics integration, the widespread practice of publishing multiple papers from the same experimental model can conceal important information about the interaction of the different factors acting towards the expression of the phenotype. In future meta-analyses on the subject, authors must consider this practice before pooling the extracted data together to avoid overestimation of the number of biological replicates.

It is crucial to ensure the highest quality standards from the research in paternal epigenetic inheritance of metabolic traits. As the prevalence of non-communicable diseases continues to increase despite all the efforts for its prevention, it is urgent to understand other mechanisms influencing the individual susceptibility to disease. The inter-, trans-, and multigenerational effects of environmental exposures transmitted by epigenetic factors likely play a relevant part in this susceptibility. More research on the subject, particularly in the paternal influence on health of the progeny, may help raise awareness beyond the scientific community, and build new policies and habits that may prevent disease. The health of future generations depends on our lifestyle choices and on a toxicant-free environment.

## Methods

### Manuscript screening and inclusion criteria

Publications were initially screened using Scopus search on the 8th December 2021, and updated on the 27th August 2023 following the reviewers’ comments. An advanced search was performed using the formula: ((TITLE-ABS-KEY (transgenerational* AND patern*) OR TITLE-ABS-KEY (transgenerational* AND male) OR TITLE-ABS-KEY (transgenerational* AND father) or TITLE-ABS-KEY (transgenerational* AND sire)).

OR (TITLE-ABS-KEY (intergenerational* AND patern*) OR TITLE-ABS-KEY (intergenerational* AND male) OR TITLE-ABS-KEY (intergenerational* AND father) or TITLE-ABS-KEY (intergenerational* AND sire)) OR (TITLE-ABS-KEY (multigenerational* AND patern*) OR TITLE-ABS-KEY (multigenerational* AND male) OR TITLE-ABS-KEY (multigenerational* AND father) or TITLE-ABS-KEY (multigenerational* AND sire))) OR KEY (epigenetic AND inheritance) AND TITLE-ABS-KEY (metabol*) AND NOT TITLE-ABS-KEY (*fish* OR drosophila OR madaka OR danio) AND NOT TITLE ("review*" OR "meta-analysis") AND PUBYEAR < 2023 AND (LIMIT-TO (DOCTYPE, "ar")). This search retrieved 1045 results (Fig. 1). Sixty-three publications were readily excluded due to their print published date being in 2023. The 982 remaining papers were then manually screened for exclusion criteria. Out of the initial manuscripts obtained through the keyword search, we excluded 64 due to their non-original research nature, and an additional 2 were excluded as they were case reports; 143 publications were excluded because the studies were not conducted in humans or other mammals; 217 exclusively studied the female factor or the maternal lineage and 263 were excluded due to inadequate transgenerational experimental design (Fig. 1a). The final selection included 295 original research papers describing multi-, inter-, or transgenerational inheritance via the paternal lineage in mammals (Additional file 2). We have excluded non-mammalian studies from our analysis as one of our primary objectives is to offer a comprehensive overview of preclinical and translational research specifically focused on paternal inheritance of acquired traits in mammals.

Papers excluded due to “inadequate transgenerational experimental design” refer to studies that do not fit any of the experimental designs of epigenetic inheritance (Fig. 1b, c). A study design commonly wrongly referred as intergenerational study are designs describing the effects of in utero exposure in the offspring. This experimental design does not evaluate any epigenetic inheritance mechanism, as the offspring is directly exposed to the same environmental stimuli as the mother [137]. However, in utero exposure studies reporting effects in the male grandoffspring meet the inclusion criteria, as they result from fathers exposed to a stimuli during embryonic development. Papers excluded due to “maternal lineage only” comprehend studies exclusively describing epigenetic inheritance along the maternal lineage and multigenerational female exposure experiments. Studies where exposed males and females are mated to produce the unexposed progeny were also excluded due to “maternal lineage,” as it is not possible to dissociate the outcomes in the progeny from the maternal or paternal lineage with this experimental setting. Nevertheless, if the breeding scheme also includes exposed male x naïve female mating pairs and the outcomes of their male offspring are described, then the paper met the inclusion criteria.

### Scientometrics analysis

Bibliographic data of the 295 selected papers was retrieved from Scopus and reports were generated using the “Analyze search” tool. Reports on author, country, institution, journals, and total publications per year were exported for downstream analysis. Authorship/co-authorship networks and paper semantics analysis were generated using VOSviewer 1.6.19 [138]. Authorship network was generated based on the joint publication count of authors with at least 3 publications and 2 co-authorships with other authors. Two semantic analyses were performed: (1) binary count of term occurrences in title, abstract, and keyword fields of selected papers and (2) co-occurrence of terms in author-defined keywords. Only terms with at least 5 occurrences were included in the analysis. Journal metrics were complemented by screening the latest InCites Journal Citation Reports (Clarivate^®^, 2023). World maps and publication trends were generated using Microsoft Office Excel 365.

### Data extraction of selected papers

Selected papers were manually screened, and their information quantitatively and qualitatively evaluated. Selected manuscripts were first segregated in human vs. animal studies. Human studies were categorized according to the design of the study as prospective or retrospective (historical). Human studies meeting the inclusion criteria were further evaluated according to The Cambridge Quality Checklists [24]. Animal studies were categorized according to the type of exposure (ancestral exposure study or gamete/embryo exposure study) and to the type of study (descriptive (associative) or mechanistic). Additionally, studies reporting the cumulative effects of environmental exposure over generations were classified as multigenerational studies.

### Multiomics integration of publications based on the same experiment

We have integrated multiple *omics* data published in 5 different papers previously published by our research group, using the same animal model of ancestral paternal exposure to HFD [90, 97, 102, 128, 129]. Two of the papers included in this analysis fit the inclusion criteria of the systematic review [90, 97] and were included in it, while the other three papers report to the same animal model (license number: 0421/000/000/2016). The publications contain testicular metabolomics, testicular lipidomics, and sperm small RNA transcriptomics on 3 generations of male mice (exposed mice – unexposed offspring – unexposed grandoffspring). To integrate multiomics data, raw peak areas of ^1^H-NMR (metabolites) and GC–MS (fatty acids), and normalized counts of RNA-seq (sncRNA) were normalized using linear regression [139]. Normalized counts were obtained using DESeq2 [140]. Multiomics integration was performed using sparse Projection of Latent Structures Discriminant Analysis (sPLS-DA), available in the mixOmics package [141]. The first 2 latent structures were estimated and the top 90% variables with highest loading in each component were selected. The methods applied for RNA-seq sequence count normalization, linear regression normalization, and multiomics integration were run in R 4.3.1 for Windows 64-bit [142].

### Supplementary Information

**Additional file 1: Table S1.** Top 10 cited papers in paternal Epigenetic Inheritance of metabolic traits in mammalians. **Table S2.** Top 10 authors considering the number of publications in paternal epigenetic inheritance of metabolic traits in mammalians. **Table S3.** Human cohort studies of paternal epigenetic inheritance.

**Additional file 2.** Full list of included studies.

## Data Availability

All data generated or analyzed during this study are included in this published article, its supplementary information files and publicly available repositories. The scripts used for the initial Scopus bibliographical search and for extracting bibliographical information of selected publications, the spreadsheets with stepwise screening of the publications, the datasets used in the “Multiomics integration” section, and the R script to process the multiomics datasets are publicly available in Figshare: https://doi.org/10.6084/m9.figshare.24421954.v1.

## Abbreviations

BPA: Bisphenol A
DDT: Dichlorodiphenyltrichloroethane
DNA: Deoxyribonucleic acid
ETH: Eidgenössische Technische Hochschule Zürich
HFD: High-fat diet
IF: Impact Factor
JCR: Journal Citation Report
KO: Knock-out (of a gene)
KI: Knock-in (of a gene)
lncRNA: Long non-coding RNA
miRNA: MicroRNA
mRNA: Messenger RNA
piRNA: Piwi RNA
tRNA: Transfer RNA
tsRNA: tRNA-derived small RNA
RNA: Ribonucleic acid
ROSI: Round Spermatid Injection
rRNA: Ribosomal RNA
rsRNA: rRNA-derived RNA
sncRNA: Small non-coding RNA
