# Supplementary material for: A systematic scientometric review of paternal inheritance of acquired metabolic traits
Source: BMC Biol. 2023 Nov 13;21:255. doi: 10.1186/s12915-023-01744-6 (PMC10641967; doi:10.1186/s12915-023-01744-6)
Supplement: Supplementary file 1 — Additional file 1: Table S1. Top 10 cited papers in paternal Epigenetic Inheritance of metabolic traits in mammalians. Table S2. Top 10 authors considering the number of publications in paternal epigenetic inheritance of metabolic traits in mammalians. Table S3. Human cohort studies of paternal epigenetic inheritance. [file 12915_2023_1744_MOESM1_ESM.docx]

**Table S1:** Top 10 cited papers in paternal Epigenetic Inheritance of metabolic traits in mammalians published before 2023 (as of August 2023). Abbreviations: DMR – Differently methylated region; N.A. – Information not available

| **Rank** | **Title** | **Author** | **Journal** | **Year** | **Number of citations** | **Design** | **Exposure -> Vehicle -> Outcomes** |
| --- | --- | --- | --- | --- | --- | --- | --- |
| **1.** | Paternally induced transgenerational environmental reprogramming of metabolic gene expression in mammals | Carone B.R., Fauquier L., Habib N., Shea J.M., Hart C.E., Li R., Bock C., Li C., Gu H., Zamore P.D., Meissner A., Weng Z., Hofmann H.A., Friedman N., Rando O.J. | Cell | 2010 | 876 | Ancestral exposure, Descriptive (associated), Non-multigenerational | Low-protein diet -> N.A. -> Body weight, serum cholesterol esters Liver DNA methylome, liver gene expression |
| **2.** | Sperm tsRNAs contribute to intergenerational inheritance of an acquired metabolic disorder | Chen Q., Yan M., Cao Z., Li X., Zhang Y., Shi J., Feng G.-H., Peng H., Zhang X., Zhang Y., Qian J., Duan E., Zhai Q., Zhou Q. | Science | 2016 | 851 | Gamete/embryo exposure, Mechanistic, Non-multigenerational | High-fat diet -> tsRNA from high-fat diet mouse sperm -> Metabolic disease, insulin sensitivity, embryo DMRs |
| **3.** | Implication of sperm RNAs in transgenerational inheritance of the effects of early trauma in mice | Gapp K., Jawaid A., Sarkies P., Bohacek J., Pelczar P., Prados J., Farinelli L., Miska E., Mansuy I.M. | Nature Neuroscience | 2014 | 830 | Gamete/embryo exposure, Mechanistic, Non-multigenerational | Stress -> sperm RNAs from traumatized mice -> Behaviour, metabolic response, embryo miRNAs |
| **4.** | Cardiovascular and diabetes mortality determined by nutrition during parents' and grandparents' slow growth period | Kaati G., Bygren L.O., Edvinsson S. | European Journal of Human Genetics | 2002 | 647 | Retrospective (historical) | Undernutrition, overnutrition -> N.A. -> Diabetes, cardiovascular disease |
| **5.** | Transgenerational inheritance of epigenetic states at the murine AxinFu allele occurs after maternal and paternal transmission | Rakyan V.K., Chong S., Champ M.E., Cuthbert P.C., Morgan H.D., Luu K.V.K., Whitelaw E. | Proceedings of the National Academy of Sciences of the United States of America | 2003 | 541 | Ancestral exposure, Mechanistic, Non-multigenerational | Axin-fused methylation -> Sperm DMR -> Kinked tail |
| **6.** | Transgenerational epigenetic programming via sperm microRNA recapitulates effects of paternal stress | Rodgers A.B., Morgan C.P., Leu N.A., Bale T.L. | Proceedings of the National Academy of Sciences of the United States of America | 2015 | 488 | Ancestral exposure, Mechanistic, Non-multigenerational | Stress -> sperm miRNAs of stressed mice -> Stress response, Embryo extracellular matrix and collagen metabolism |
| **7.** | Transgenerational effects of prenatal exposure to the Dutch famine on neonatal adiposity and health in later life | Painter R.C., Osmond C., Gluckman P., Hanson M., Phillips D.I.W., Roseboom T.J. | BJOG: An International Journal of Obstetrics and Gynaecology | 2008 | 468 | Prospective | Undernutrition -> N.A. -> Body Weight, BMI, metabolic disease |
| **8.** | Paternal obesity initiates metabolic disturbances in two generations of mice with incomplete penetrance to the F2 generation and alters the transcriptional profile of testis and sperm microRNA content | Fullston T., Teague E.M.C.O., Palmer N.O., Deblasio M.J., Mitchell M., Corbett M., Print C.G., Owens J.A., Lane M. | FASEB Journal | 2013 | 424 | Ancestral exposure, Ecological, Non-multigenerational | High-fat diet -> Sperm miRNAs, DNA methylation -> body weight, insulin sensitivity, testicular and sperm transcriptome, sperm methylation rate |
| **9.** | Epigenetic transgenerational actions of vinclozolin on promoter regions of the sperm epigenome | Guerrero-Bosagna C., Settles M., Lucker B., Skinner M.K. | PLoS ONE | 2010 | 336 | Gamete/embryo exposure, Descriptive (associated), Non-multigenerational | Vinclozolin -> N.A. -> Sperm DMRs and DNA methylation rate, promoter copy number |
| **10.** | Disruption of histone methylation in developing sperm impairs offspring health transgenerationally | Siklenka K., Erkek S., Godmann M., Lambrot R., McGraw S., Lafleur C., Cohen T., Xia J., Suderman M., Hallett M., Trasler J., Peters A.H.F.M., Kimmins S. | Science | 2015 | 334 | Ancestral exposure, Descriptive (associated), Non-multigenerational | High-fat diet -> Sperm histone methylation -> Testicular histone methylase expression, sperm CpG islands methylation |

**Table S2:** Top 10 authors considering the number of publications in paternal epigenetic inheritance of metabolic traits in mammalians (As of December 2021).

| **Rank** | **Author** | **Institution** | **Country** | **Publications** |
| --- | --- | --- | --- | --- |
| **1.** | Skinner, M.K. | Washington State University Pullman | United States | 23 |
| **2.** | Beck, D. | Washington State University Pullman | United States | 14 |
| **3.** | Nilsson, E. | Washington State University Pullman | United States | 10 |
| **4.** | Ben Maamar, M. | Washington State University Pullman | United States | 8 |
| **5.** | Hannan, A.J. | The Florey | Australia | 7 |
|  | Mansuy, I.M. | Universität Zürich | Switzerland | 7 |
|  | Sadler-Riggleman, I. | Washington State University Pullman | United States | 7 |
| **8.** | Nilsson, E.E. | Washington State University Pullman | United States | 6 |
|  | Pang, T.Y. | The Florey | Australia | 6 |
| **10.** | Crews, D. | The University of Texas at Austin | United States | 5 |

**Table S3:** Human cohort studies of paternal epigenetic inheritance and their Cambridge Quality Checklist scores.

|  |  |  |  | **The Cambridge Quality Checklists** | | | |
| --- | --- | --- | --- | --- | --- | --- | --- |
| **Title** | **Authors** | **Journal** | **Year** | **Correlates** | **Risk factor** | **Causal risk factor** | **Total score** |
| Consequences of parental preconceptional irradiation: Endocrine-metabolic pathology in offspring | Sosnina S.F.; Okatenko P.V.; Sokolnikov M.E. | Radiatsionnaya Gygiena | 2022 | 2 | 2 | 4 | **8** |
| Parental preconception BMI trajectories from childhood to adolescence and asthma in the future offspring | Bowatte G.; Bui D.S.; Priyankara S.; Lowe A.J.; Perret J.L.; Lodge C.J.; Hamilton G.S.; Erbas B.; Thomas P.; Thompson B.; Schlünssen V.; Martino D.; Holloway J.W.; Svanes C.; Abramson M.J.; Walters E.H.; Dharmage S.C. | Journal of Allergy and Clinical Immunology | 2022 | 1 | 2 | 3 | **6** |
| Famine and Trajectories of Body Mass Index, Waist Circumference, and Blood Pressure in Two Generations: Results from the CHNS from 1993-2015 | Li J.; Yang Q.; An R.; Sesso H.D.; Zhong V.W.; Chan K.H.K.; Madsen T.E.; Papandonatos G.D.; Zheng T.; Wu W.-C.; Song Y.; Yu X.; Liu S. | Hypertension | 2022 | 5 | 2 | 5 | **12** |
| Investigation of the Causal Association between Long-Chain n-6 Polyunsaturated Fatty Acid Synthesis and the Risk of Type 2 Diabetes: A Mendelian Randomization Analysis | Zulyniak M.A., Fuller H., Iles M.M. | Lifestyle Genomics | 2020 | 3 | 2 | 2 | **7** |
| Age-associated sperm DNA methylation patterns do not directly persist trans-generationally | Jenkins T.G., James E.R., Aston K.I., Salas-Huetos A., Pastuszak A.W., Smith K.R., Hanson H.A., Hotaling J.M., Carrell D.T. | Epigenetics and Chromatin | 2019 | 2 | 3 | 5 | **10** |
| Grandmothers’ smoking in pregnancy is associated with a reduced prevalence of early-onset myopia | Williams C., Suderman M., Guggenheim J.A., Ellis G., Gregory S., Iles-Caven Y., Northstone K., Golding J., Pembrey M. | Scientific Reports | 2019 | 1 | 3 | 5 | **9** |
| DNA methylation from germline cells in veterans with PTSD | Mehta D., Pelzer E.S., Bruenig D., Lawford B., McLeay S., Morris C.P., Gibson J.N., Young R.M., Voisey J., Harvey W., Romaniuk M., Crawford D., Colquhoun D., Dwyer M., Gibson J., O'Sullivan R., Cooksley G., Strakosch C., Thomson R., PTSD Initiative | Journal of Psychiatric Research | 2019 | 0 | 3 | 5 | **8** |
| Reduced Sensitivity to Thyroid Hormone as a Transgenerational Epigenetic Marker Transmitted Along the Human Male Line | Anselmo J., Scherberg N.H., Dumitrescu A.M., Refetoff S. | Thyroid | 2019 | 1 | 3 | 2 | **6** |
| Parent’s cardiorespiratory fitness, body mass, and chronic disease status is associated with metabolic syndrome in young adults: A preliminary study | Nolan P.B., Carrick-Ranson G., Stinear J.W., Reading S.A., Dalleck L.C. | International Journal of Environmental Research and Public Health | 2019 | 1 | 2 | 5 | **8** |
| Multigenerational metabolic profiling in the Michigan PBB registry | Walker D.I., Marder M.E., Yano Y., Terrell M., Liang Y., Barr D.B., Miller G.W., Jones D.P., Marcus M., Pennell K.D. | Environmental Research | 2019 | 1 | 3 | 2 | **6** |
| Parental non-alcoholic fatty liver disease increases risk of non-alcoholic fatty liver disease in offspring | Long M.T., Gurary E.B., Massaro J.M., Ma J., Hoffmann U., Chung R.T., Benjamin E.J., Loomba R. | Liver International | 2019 | 1 | 3 | 5 | **9** |
| Characterization of the contribution of shared environmental and genetic factors to metabolic syndrome methylation heritability and familial correlations | Fernández-Rhodes L.; Howard A.G.; Tao R.; Young K.L.; Graff M.; Aiello A.E.; North K.E.; Justice A.E. | BMC Genetics | 2018 | 3 | 2 | 3 | **8** |
| Prenatal exposure to famine and the development of hyperglycemia and type 2 diabetes in adulthood across consecutive generations: A population-based cohort study of families in Suihua, China | Li J., Liu S., Li S., Feng R., Na L., Chu X., Wu X., Niu Y., Sun Z., Han T., Deng H., Meng X., Xu H., Zhang Z., Qu Q., Zhang Q., Li Y., Sun C. | American Journal of Clinical Nutrition | 2017 | 2 | 2 | 5 | **9** |
| Longer duration and earlier age of onset of paternal betel chewing and smoking increase metabolic syndrome risk in human offspring, independently, in a community-based screening program in Taiwan | Yen A.M.-F., Boucher B.J., Chiu S.Y.-H., Fann J.C.-Y., Chen S.L.-S., Huang K.-C., Chen H.-H. | Circulation | 2016 | 2 | 2 | 5 | **9** |
| Analysis of a four generation family reveals the widespread sequence-dependent maintenance of allelic DNA methylation in somatic and germ cells | Tang A., Huang Y., Li Z., Wan S., Mou L., Yin G., Li N., Xie J., Xia Y., Li X., Luo L., Zhang J., Chen S., Wu S., Sun J., Sun X., Jiang Z., Chen J., Li Y., Wang J., Wang J., Cai Z., Gui Y. | Scientific Reports | 2016 | 0 | 1 | 1 | **2** |
| The role of life-course socioeconomic and lifestyle factors in the intergenerational transmission of the metabolic syndrome: Results from the LifeLines Cohort Study | Klijs B., Angelini V., Mierau J.O., Smidt N. | International Journal of Epidemiology | 2016 | 3 | 3 | 5 | **11** |
| Blood pressure abnormalities in adults born moderately preterm and their children | Mathai S., Derraik J.G.B., Cutfield W.S., Dalziel S.R., Harding J.E., Biggs J.B., Jefferies C., Hofman P.L. | International Journal of Cardiology | 2015 | 0 | 3 | 5 | **8** |
| Prepubertal start of father's smoking and increased body fat in his sons: further characterisation of paternal transgenerational responses | Northstone K., Golding J., Davey Smith G., Miller L.L., Pembrey M. | European journal of human genetics : EJHG | 2014 | 2 | 3 | 5 | **10** |
| Cardiovascular diseases in grandparents and the risk of congenital heart diseases in grandchildren | Wijnands K.P.J., Obermann-Borst S.A., Sijbrands E.J.G., Wildhagen M.F., Helbing W.A., Steegers-Theunissen R.P.M. | Journal of Developmental Origins of Health and Disease | 2014 | 2 | 3 | 5 | **10** |
| Transgenerational effects of prenatal exposure to the 1944-45 Dutch famine | Veenendaal M.V.E., Painter R.C., De Rooij S.R., Bossuyt P.M.M., Van Der Post J.A.M., Gluckman P.D., Hanson M.A., Roseboom T.J. | BJOG: An International Journal of Obstetrics and Gynaecology | 2013 | 1 | 3 | 5 | **9** |
| Transgenerational effects of prenatal exposure to the Dutch famine on neonatal adiposity and health in later life | Painter R.C., Osmond C., Gluckman P., Hanson M., Phillips D.I.W., Roseboom T.J. | BJOG: An International Journal of Obstetrics and Gynaecology | 2008 | 1 | 3 | 5 | **9** |
| Paternal smoking is associated with a decreased prevalence of type 1 diabetes mellitus among offspring in two national British birth cohort studies (NCDS and BCS70) | Toschke A.M., Ehlin A., Koletzko B., Montgomery S.M. | Journal of Perinatal Medicine | 2007 | 4 | 3 | 5 | **12** |
| Transgenerational effects of betel-quid chewing on the development of the metabolic syndrome in the Keelung Community-based Integrated Screening Program | Chen T.H.-H., Chiu Y.-H., Boucher B.J. | American Journal of Clinical Nutrition | 2006 | 2 | 2 | 5 | **9** |
| Cardiovascular and diabetes mortality determined by nutrition during parents' and grandparents' slow growth period | Kaati G., Bygren L.O., Edvinsson S. | European Journal of Human Genetics | 2002 | 2 | 3 | 5 | **10** |
| Differential sensitivity to alcohol reinforcement in groups of men at risk for distinct alcoholism subtypes | Conrod P.J., Pihl R.O., Vassileva J. | Alcoholism: Clinical and Experimental Research | 1998 | 1 | 2 | 5 | **8** |
