# Supplementary material for: A systematic scientometric review of paternal inheritance of acquired metabolic traits
Source: BMC Biol. 2023 Nov 13;21:255. doi: 10.1186/s12915-023-01744-6 (PMC10641967; doi:10.1186/s12915-023-01744-6)
Supplement: Supplementary file 2 — Additional file 2. Full list of included studies. [file 12915_2023_1744_MOESM2_ESM.docx]

**Full list of included studies**

**Studies included in systematic review and scientometrics analysis**

1. Abbott CW, Rohac DJ, Bottom RT, Patadia S, Huffman KJ: Prenatal ethanol exposure and neocortical development: A transgenerational model of FASD. Cereb Cortex 2018, 28(8):2908-2921.

2. Abrantes MA, Valencia AM, Bany-Mohammed F, Aranda JV, Beharry KD: Intergenerational Influence of Antenatal Betamethasone on Growth, Growth Factors, and Neurological Outcomes in Rats. Reproductive Sciences 2020, 27(1):418-431.

3. Acharya KS, Schrott R, Grenier C, Huang Z, Holloway Z, Hawkey A, Levin ED, Murphy SK: Epigenetic alterations in cytochrome P450 oxidoreductase (Por) in sperm of rats exposed to tetrahydrocannabinol (THC). Sci Rep 2020, 10(1).

4. Adedeji TG, Fasanmade A, Olapade-Olaopa E: Multigenerational effects of dietary macronutrient intake on the metabolic phenotype of male Wistar rats. Nutrition 2019, 58:125-133.

5. Adegoke EO, Rahman MS, Amjad S, Pang WK, Ryu DY, Park YJ, Pang MG: Bisphenol A damages testicular junctional proteins transgenerationally in mice. Environmental Pollution 2022, 302.

6. Aizawa S, Tochihara A, Yamamuro Y: Paternal high-fat diet alters triglyceride metabolism-related gene expression in liver and white adipose tissue of male mouse offspring. Biochem Biophys Rep 2022, 31.

7. Al-Griw MA, Alghazeer RO, Salama NM, Lwaleed BA, Eskandrani AA, Alansari WS, Alnajeebi AM, Babteen NA, Shamlan G, Elnfati AH: Paternal bisphenol A exposure induces testis and sperm pathologies in mice offspring: Possibly due to oxidative stress? Saudi J Biol Sci 2021, 28(1):948-955.

8. Alshanbayeva A, Tanwar DK, Roszkowski M, Manuella F, Mansuy IM: Early life stress affects the miRNA cargo of epididymal extracellular vesicles in mouse. Biology of Reproduction 2021, 105(3):593-602.

9. Alves-Wagner AB, Kusuyama J, Nigro P, Ramachandran K, Makarewicz N, Hirshman MF, Goodyear LJ: Grandmaternal exercise improves metabolic health of second-generation offspring. Mol Metab 2022, 60.

10. Amri J, Sadegh M, Moulaei N, Palizvan MR: Transgenerational modification of hippocampus TNF-α and S100B levels in the offspring of rats chronically exposed to morphine during adolescence. Am J Drug Alcohol Abuse 2018, 44(1):95-102.

11. An T, Zhang T, Teng F, Zuo JC, Pan YY, Liu YF, Miao JN, Gu YJ, Yu N, Zhao DD et al: Long non-coding RNAs could act as vectors for paternal heredity of high fat diet-induced obesity. Oncotarget 2017, 8(29):47876-47889.

12. Anderson LM, Riffle L, Wilson R, Travlos GS, Lubomirski MS, Alvord WG: Preconceptional fasting of fathers alters serum glucose in offspring of mice. Nutrition 2006, 22(3):327-331.

13. Anselmo J, Scherberg NH, Dumitrescu AM, Refetoff S: Reduced Sensitivity to Thyroid Hormone as a Transgenerational Epigenetic Marker Transmitted Along the Human Male Line. Thyroid 2019, 29(6):778-782.

14. Aoued HS, Sannigrahi S, Doshi N, Morrison FG, Linsenbaum H, Hunter SC, Walum H, Baman J, Yao B, Jin P et al: Reversing Behavioral, Neuroanatomical, and Germline Influences of Intergenerational Stress. Biol Psychiatry 2019, 85(3):248-256.

15. Aoued HS, Sannigrahi S, Hunter SC, Doshi N, Sathi ZS, Chan AWS, Walum H, Dias BG: Proximate causes and consequences of intergenerational influences of salient sensory experience. Genes Brain Behav 2020, 19(4).

16. Baptissart M, Sèdes L, Holota H, Thirouard L, Martinot E, de Haze A, Rouaisnel B, Caira F, Beaudoin C, Volle DH: Multigenerational impacts of bile exposure are mediated by TGR5 signaling pathways. Sci Rep 2018, 8(1).

17. Beck D, Ben Maamar M, Skinner MK: Integration of sperm ncRNA-directed DNA methylation and DNA methylation-directed histone retention in epigenetic transgenerational inheritance. Epigenetics Chromatin 2021, 14(1).

18. Beck D, Nilsson EE, Ben Maamar M, Skinner MK: Environmental induced transgenerational inheritance impacts systems epigenetics in disease etiology. Sci Rep 2022, 12(1).

19. Ben Maamar M, Beck D, Nilsson E, McCarrey JR, Skinner MK: Developmental origins of transgenerational sperm histone retention following ancestral exposures. Developmental Biology 2020, 465(1):31-45.

20. Ben Maamar M, Beck D, Nilsson EE, Kubsad D, Skinner MK: Epigenome-wide association study for glyphosate induced transgenerational sperm DNA methylation and histone retention epigenetic biomarkers for disease. Epigenetics 2021, 16(10):1150-1167.

21. Ben Maamar M, Nilsson E, Thorson JLM, Beck D, Skinner MK: Transgenerational disease specific epigenetic sperm biomarkers after ancestral exposure to dioxin. Environ Res 2021, 192.

22. Ben Maamar M, Sadler-Riggleman I, Beck D, Skinner MK: Epigenetic Transgenerational Inheritance of Altered Sperm Histone Retention Sites. Sci Rep 2018, 8(1).

23. Benito E, Kerimoglu C, Ramachandran B, Pena-Centeno T, Jain G, Stilling RM, Islam MR, Capece V, Zhou Q, Edbauer D et al: RNA-Dependent Intergenerational Inheritance of Enhanced Synaptic Plasticity after Environmental Enrichment. Cell Reports 2018, 23(2):546-554.

24. Bodden C, Pang TY, Feng Y, Mridha F, Kong G, Li S, Watt MJ, Reichelt AC, Hannan AJ: Intergenerational effects of a paternal Western diet during adolescence on offspring gut microbiota, stress reactivity, and social behavior. FASEB journal: official publication of the Federation of American Societies for Experimental Biology 2022, 36(1).

25. Bomans K, Schenz J, Tamulyte S, Schaack D, Weigand MA, Uhle F: Paternal sepsis induces alterations of the sperm methylome and dampens offspring immune responses-an animal study. Clinical Epigenetics 2018, 10(1).

26. Bönisch C, Irmler M, Brachthäuser L, Neff F, Bamberger MT, Marschall S, Hrabě de Angelis M, Beckers J: Dexamethasone treatment alters insulin, leptin, and adiponectin levels in male mice as observed in DIO but does not lead to alterations of metabolic phenotypes in the offspring. Mammalian Genome 2016, 27(1-2):17-28.

27. Bordoni L, Nasuti C, Di Stefano A, Marinelli L, Gabbianelli R: Epigenetic Memory of Early-Life Parental Perturbation: Dopamine Decrease and DNA Methylation Changes in Offspring. Oxidative Med Cell Longevity 2019, 2019.

28. Borges CDS, Pacheco TL, da Silva KP, Fernandes FH, Gregory M, Pupo AS, Salvadori DMF, Cyr DG, Kempinas WDG: Betamethasone causes intergenerational reproductive impairment in male rats. Reprod Toxicol 2017, 71:108-117.

29. Boscardin C, Manuella F, Mansuy IM: Paternal transmission of behavioural and metabolic traits induced by postnatal stress to the 5th generation in mice. Environmental Epigenetics 2022, 8(1).

30. Bowatte G, Bui DS, Priyankara S, Lowe AJ, Perret JL, Lodge CJ, Hamilton GS, Erbas B, Thomas P, Thompson B et al: Parental preconception BMI trajectories from childhood to adolescence and asthma in the future offspring. J Allergy Clin Immunol 2022, 150(1):67-74.e30.

31. Brass KE, Herndon N, Gardner SA, Grindstaff JL, Campbell P: Intergenerational effects of paternal predator cue exposure on behavior, stress reactivity, and neural gene expression. Hormones and behavior 2020, 124.

32. Braunschweig M, Jagannathan V, Gutzwiller A, Bee G: Investigations on transgenerational epigenetic response down the male line in F2 pigs. PLoS ONE 2012, 7(2).

33. Brieño-Enríquez MA, García-López J, Cárdenas DB, Guibert S, Cleroux E, Děd L, Hourcade JDD, Pěknicová J, Weber M, Del Mazo J: Exposure to endocrine disruptor induces transgenerational epigenetic deregulation of microRNAs in primordial germ cells. PLoS ONE 2015, 10(4).

34. Brulport A, Le Corre L, Maquart G, Barbet V, Dastugue A, Severin I, Vaiman D, Chagnon MC: Multigenerational study of the obesogen effects of bisphenol S after a perinatal exposure in C57BL6/J mice fed a high fat diet. Environmental Pollution 2021, 270.

35. Brulport A, Lencina C, Chagnon MC, Le Corre L, Guzylack-Piriou L: Transgenerational effects on intestinal inflammation status in mice perinatally exposed to bisphenol S. Chemosphere 2021, 262.

36. Cambiasso MY, Gotfryd L, Stinson MG, Birolo S, Salamone G, Romanato M, Calvo JC, Fontana VA: Paternal alcohol consumption has intergenerational consequences in male offspring. Journal of Assisted Reproduction and Genetics 2022, 39(2):441-459.

37. Cao J, Chen Y, Xia X, Qu H, Ao Y, Wang H: Intergenerational genetic programming mechanism and sex differences of the adrenal corticosterone synthesis dysfunction in offspring induced by prenatal ethanol exposure. Toxicol Lett 2021, 351:78-88.

38. Carone BR, Fauquier L, Habib N, Shea JM, Hart CE, Li R, Bock C, Li C, Gu H, Zamore PD et al: Paternally induced transgenerational environmental reprogramming of metabolic gene expression in mammals. Cell 2010, 143(7):1084-1096.

39. Cartier J, Smith T, Thomson JP, Rose CM, Khulan B, Heger A, Meehan RR, Drake AJ: Investigation into the role of the germline epigenome in the transmission of glucocorticoid-programmed effects across generations. Genome biology 2018, 19(1).

40. Chamorro-Garcia R, Diaz-Castillo C, Shoucri BM, Käch H, Leavitt R, Shioda T, Blumberg B: Ancestral perinatal obesogen exposure results in a transgenerational thrifty phenotype in mice. Nat commun 2017, 8(1).

41. Chamorro-García R, Poupin N, Tremblay-Franco M, Canlet C, Egusquiza R, Gautier R, Jouanin I, Shoucri BM, Blumberg B, Zalko D: Transgenerational metabolomic fingerprints in mice ancestrally exposed to the obesogen TBT. Environ Int 2021, 157.

42. Chang RC, Wang H, Bedi Y, Golding MC: Preconception paternal alcohol exposure exerts sex-specific effects on offspring growth and long-term metabolic programming. Epigenetics Chromatin 2019, 12(1).

43. Chen J, Wu S, Wen S, Shen L, Peng J, Yan C, Cao X, Zhou Y, Long C, Lin T et al: The mechanism of environmental endocrine disruptors (DEHP) induces epigenetic transgenerational inheritance of cryptorchidism. PLoS ONE 2015, 10(6).

44. Chen Q, Yan M, Cao Z, Li X, Zhang Y, Shi J, Feng G-h, Peng H, Zhang X, Zhang Y et al: Sperm tsRNAs contribute to intergenerational inheritance of an acquired metabolic disorder. Science 2016, 351(6271):397-400.

45. Chen THH, Chiu YH, Boucher BJ: Transgenerational effects of betel-quid chewing on the development of the metabolic syndrome in the Keelung Community-based Integrated Screening Program. Am J Clin Nutr 2006, 83(3):688-692.

46. Cheng RYS, Alvord WG, Powell D, Kasprzak KS, Anderson LM: Increased serum corticosterone and glucose in offspring of chromium(III)-treated male mice. Environ Health Perspect 2002, 110(8):801-804.

47. Chleilat F, Schick A, Deleemans JM, Ma K, Alukic E, Wong J, Noye Tuplin EW, Nettleton JE, Reimer RA: Paternal high protein diet modulates body composition, insulin sensitivity, epigenetics, and gut microbiota intergenerationally in rats. FASEB journal: official publication of the Federation of American Societies for Experimental Biology 2021, 35(9).

48. Chleilat F, Schick A, Deleemans JM, Reimer RA: Paternal methyl donor supplementation in rats improves fertility, physiological outcomes, gut microbial signatures and epigenetic markers altered by high fat/high sucrose diet. International Journal of Molecular Sciences 2021, 22(2):1-19.

49. Chleilat F, Schick A, Reimer RA: Microbiota changes in fathers consuming a high prebiotic fiber diet have minimal effects on male and female offspring in rats. Nutrients 2021, 13(3):1-15.

50. Choi CS, Gonzales EL, Kim KC, Yang SM, Kim JW, Mabunga DF, Cheong JH, Han SH, Bahn GH, Shin CY: The transgenerational inheritance of autism-like phenotypes in mice exposed to valproic acid during pregnancy. Sci Rep 2016, 6.

51. Cissé YM, Chan JC, Nugent BM, Banducci C, Bale TL: Brain and placental transcriptional responses as a readout of maternal and paternal preconception stress are fetal sex specific. Placenta 2020, 100:164-170.

52. Conrod PJ, Pihl RO, Vassileva J: Differential Sensitivity to Alcohol Reinforcement in Groups of Men at Risk for Distinct Alcoholism Subtypes. Alcoholism: Clinical and Experimental Research 1998, 22(3):585-597.

53. Constantinof A, Moisiadis VG, Kostaki A, Szyf M, Matthews SG: Antenatal Glucocorticoid Exposure Results in Sex-Specific and Transgenerational Changes in Prefrontal Cortex Gene Transcription that Relate to Behavioural Outcomes. Sci Rep 2019, 9(1).

54. Costa-Júnior JM, Ferreira SM, Kurauti MA, Bernstein DL, Ruano EG, Kameswaran V, Schug J, Freitas-Dias R, Zoppi CC, Boschero AC et al: Paternal exercise improves the metabolic health of offspring via epigenetic modulation of the germline. International Journal of Molecular Sciences 2022, 23(1).

55. Crews D, Gillette R, Scarpino SV, Manikkam M, Savenkova MI, Skinner MK: Epigenetic transgenerational inheritance of altered stress responses. P Natl Acad Sci USA 2012, 109(23):9143-9148.

56. Crisóstomo L, Bourgery M, Rato L, Raposo JF, Batterham RL, Kotaja N, Alves MG: Testicular “inherited metabolic memory” of ancestral high-fat diet is associated with sperm sncRNA content. Biomedicines 2022, 10(4):909.

57. Crisóstomo L, Jarak I, Rato LP, Raposo JF, Batterham RL, Oliveira PF, Alves MG: Inheritable testicular metabolic memory of high-fat diet causes transgenerational sperm defects in mice. Sci Rep 2021, 11(1):9444.

58. Dabeer S, Afjal MA, Ahmad S, Fatima M, Habib H, Parvez S, Raisuddin S: Transgenerational effect of parental obesity and chronic parental bisphenol A exposure on hormonal profile and reproductive organs of preadolescent Wistar rats of F1 generation: A one-generation study. Hum Exp Toxicol 2020, 39(1):59-76.

59. Dai J, Wang Z, Xu W, Zhang M, Zhu Z, Zhao X, Zhang D, Nie D, Wang L, Qiao Z: Paternal nicotine exposure defines different behavior in subsequent generation via hyper-methylation of mmu-miR-15b. Sci Rep 2017, 7(1).

60. Dai YJ, Wu DC, Feng B, Chen B, Tang YS, Jin MM, Zhao HW, Dai HB, Wang Y, Chen Z: Prolonged febrile seizures induce inheritable memory deficits in rats through DNA methylation. CNS Neurosci Ther 2019, 25(5):601-611.

61. de Castro Barbosa T, Alm PS, Krook A, Barrès R, Zierath JR: Paternal high-fat diet transgenerationally impacts hepatic immunometabolism. FASEB journal: official publication of the Federation of American Societies for Experimental Biology 2019, 33(5):6269-6280.

62. de Castro Barbosa T, Ingerslev LR, Alm PS, Versteyhe S, Massart J, Rasmussen M, Donkin I, Sjögren R, Mudry JM, Vetterli L et al: High-fat diet reprograms the epigenome of rat spermatozoa and transgenerationally affects metabolism of the offspring. Mol Metab 2016, 5(3):184-197.

63. de la Rocha C, Rodríguez-Ríos D, Ramírez-Chávez E, Molina-Torres J, Flores-Sierra JJ, Orozco-Castellanos LM, Galván-Chía JP, Sánchez AV, Zaina S, Lund G: Cumulative Metabolic and Epigenetic Effects of Paternal and/or Maternal Supplementation with Arachidonic Acid across Three Consecutive Generations in Mice. Cells 2022, 11(6).

64. de Sousa Neto IV, Prestes J, Pereira GB, Almeida JA, Ramos GV, de Souza FHV, de Souza PEN, Tibana RA, Franco OL, Durigan JLQ et al: Protective role of intergenerational paternal resistance training on fibrosis, inflammatory profile, and redox status in the adipose tissue of rat offspring fed with a high-fat diet. Life Sciences 2022, 295.

65. De Sousa Neto IV, Tibana RA, Prestes J, De Oliveira Da Silva LG, Almeida JA, Franco OL, De Oliveira EM, Voltarelli FA, Durigan JLQ, De Sousa MV et al: Paternal Resistance Training Induced Modifications in the Left Ventricle Proteome Independent of Offspring Diet. Oxidative Med Cell Longevity 2020, 2020.

66. Deng Y, Chen H, Huang Y, Wang Q, Chen W, Chen D: Polystyrene Microplastics Affect the Reproductive Performance of Male Mice and Lipid Homeostasis in Their Offspring. Environ Sci Techno Lett 2022, 9(9):752-757.

67. Derouiche L, Keller M, Duittoz AH, Pillon D: Developmental exposure to Ethinylestradiol affects transgenerationally sexual behavior and neuroendocrine networks in male mice. Sci Rep 2015, 5.

68. Desesso JM, Scialli AR, White TEK, Breckenridge CB: Multigeneration reproduction and male developmental toxicity studies on atrazine in rats. Birth Defects Res Part B Dev Reprod Toxicol 2014, 101(3):237-253.

69. Diaz J, Taylor EM: Abnormally High Nourishment During Sensitive Periods Results in Body Weight Changes Across Generations. Obesity Research 1998, 6(5):368-374.

70. Ding T, Mokshagundam S, Rinaudo PF, Osteen KG, Bruner-Tran KL: Paternal developmental toxicant exposure is associated with epigenetic modulation of sperm and placental Pgr and Igf2 in a mouse model. Biology of Reproduction 2018, 99(4):864-876.

71. Dion V, Lin Y, Hubert Jr L, Waterland RA, Wilson JH: Dnmt1 deficiency promotes CAG repeat expansion in the mouse germline. Human Molecular Genetics 2008, 17(9):1306-1317.

72. Drake AJ, Liu L, Kerrigan D, Meehan RR, Seckl JR: Multigenerational programming in the glucocorticoid programmed rat is associated with generation-specific and parent of origin effects. Epigenetics 2011, 6(11):1334-1343.

73. Drobná Z, Henriksen AD, Wolstenholme JT, Montiel C, Lambeth PS, Shang S, Harris EP, Zhou C, Flaws JA, Adli M, Rissman EF: Transgenerational effects of bisphenol a on gene expression and DNA methylation of imprinted genes in brain. Endocrinology 2018, 159(1):132-144.

74. Dunn GA, Bale TL: Maternal high-fat diet effects on third-generation female body size via the paternal lineage. Endocrinology 2011, 152(6):2228-2236.

75. Eaton SA, Aiken AJ, Young PE, Ho JWK, Cropley JE, Suter CM: Maternal obesity heritably perturbs offspring metabolism for three generations without serial programming. International Journal of Obesity 2018, 42(4):911-914.

76. Farah Naquiah MZ, James RJ, Suratman S, Lee LS, Mohd Hafidz MI, Salleh MZ, Teh LK: Transgenerational effects of paternal heroin addiction on anxiety and aggression behavior in male offspring. Behav Brain Funct 2016, 12(1).

77. Federici F, Magaraki A, Wassenaar E, van Veen-Buurman CJH, van de Werken C, Baart EB, Laven JSE, Grootegoed JA, Gribnau J, Baarends WM: Round Spermatid Injection Rescues Female Lethality of a Paternally Inherited Xist Deletion in Mouse. PLoS Genetics 2016, 12(10).

78. Fennell KA, Busby RGG, Li S, Bodden C, Stanger SJ, Nixon B, Short AK, Hannan AJ, Pang TY: Limitations to intergenerational inheritance: subchronic paternal stress preconception does not influence offspring anxiety. Sci Rep 2020, 10(1).

79. Ferey JLA, Boudoures AL, Reid M, Drury A, Scheaffer S, Modi Z, Kovacs A, Pietka T, DeBosch BJ, Thompson MD et al: A maternal high-fat, high-sucrose diet induces transgenerational cardiac mitochondrial dysfunction independently of maternal mitochondrial inheritance. Am J Physiol Heart Circ Physiol 2019, 316(5):H1202-H1210.

80. Fernández-Rhodes L, Howard AG, Tao R, Young KL, Graff M, Aiello AE, North KE, Justice AE: Characterization of the contribution of shared environmental and genetic factors to metabolic syndrome methylation heritability and familial correlations 06 Biological Sciences 0604 Genetics. BMC Genet 2018, 19.

81. Freitas-Dias R, Lima TI, Costa-Junior JM, Gonçalves LM, Araujo HN, Paula FMM, Santos GJ, Branco RCS, Ou K, Kaestner KH et al: Offspring from trained male mice inherit improved muscle mitochondrial function through PPAR co-repressor modulation. Life Sciences 2022, 291.

82. Fullston T, Teague EMCO, Palmer NO, Deblasio MJ, Mitchell M, Corbett M, Print CG, Owens JA, Lane M: Paternal obesity initiates metabolic disturbances in two generations of mice with incomplete penetrance to the F2 generation and alters the transcriptional profile of testis and sperm microRNA content. FASEB journal: official publication of the Federation of American Societies for Experimental Biology 2013, 27(10):4226-4243.

83. Gao ZY, Chen TY, Yu TT, Zhang LP, Zhao SJ, Gu XY, Pan Y, Kong LD: Cinnamaldehyde prevents intergenerational effect of paternal depression in mice via regulating GR/miR-190b/BDNF pathway. Acta Pharmacol Sin 2022, 43(8):1955-1969.

84. Gapp K, Bohacek J, Grossmann J, Brunner AM, Manuella F, Nanni P, Mansuy IM: Potential of environmental enrichment to prevent transgenerational effects of paternal trauma. Neuropsychopharmacology 2016, 41(11):2749-2758.

85. Gapp K, Corcoba A, van Steenwyk G, Mansuy IM, Duarte JMN: Brain metabolic alterations in mice subjected to postnatal traumatic stress and in their offspring. J Cereb Blood Flow Metab 2017, 37(7):2423-2432.

86. Gapp K, Jawaid A, Sarkies P, Bohacek J, Pelczar P, Prados J, Farinelli L, Miska E, Mansuy IM: Implication of sperm RNAs in transgenerational inheritance of the effects of early trauma in mice. Nature neuroscience 2014, 17(5):667-669.

87. Garcia-Dominguez X, Marco-Jiménez F, Peñaranda DS, Diretto G, García-Carpintero V, Cañizares J, Vicente JS: Long-term and transgenerational phenotypic, transcriptional and metabolic effects in rabbit males born following vitrified embryo transfer. Sci Rep 2020, 10(1).

88. Gely-Pernot A, Hao C, Legoff L, Multigner L, D'Cruz SC, Kervarrec C, Jégou B, Tevosian S, Smagulova F: Gestational exposure to chlordecone promotes transgenerational changes in the murine reproductive system of males. Sci Rep 2018, 8(1).

89. Gill WD, Burgess KC, Vied C, Brown RW: Transgenerational evidence of increases in dopamine D2 receptor sensitivity in rodents: Impact on sensorimotor gating, the behavioral response to nicotine and BDNF. J Psychopharmacol 2021, 35(10):1188-1203.

90. Gillette R, Miller-Crews I, Nilsson EE, Skinner MK, Gore AC, Crews D: Sexually dimorphic effects of ancestral exposure to vinclozolin on stress reactivity in rats. Endocrinology (United States) 2014, 155(10):3853-3866.

91. Gillette R, Son MJ, Ton L, Gore AC, Crews D: Passing experiences on to future generations: endocrine disruptors and transgenerational inheritance of epimutations in brain and sperm. Epigenetics 2018, 13(10-11):1106-1126.

92. Goldberg LR, Zeid D, Kutlu MG, Cole RD, Lallai V, Sebastian A, Albert I, Fowler CD, Parikh V, Gould TJ: Paternal nicotine enhances fear memory, reduces nicotine administration, and alters hippocampal genetic and neural function in offspring. Addict Biol 2021, 26(1).

93. Gong Y, Xue Y, Li X, Zhang Z, Zhou W, Marcolongo P, Benedetti A, Mao S, Han L, Ding G, Sun Z: Inter- and Transgenerational Effects of Paternal Exposure to Inorganic Arsenic. Adv Sci 2021, 8(7).

94. Gonzalez-Bulnes A, Astiz S, Ovilo C, Lopez-Bote CJ, Sanchez-Sanchez R, Perez-Solana ML, Torres-Rovira L, Ayuso M, Gonzalez J: Early-postnatal changes in adiposity and lipids profile by transgenerational developmental programming in swine with obesity/leptin resistance. Journal of Endocrinology 2014, 223(1):M17-M29.

95. Gore AC, Thompson LM, Bell M, Mennigen JA: Transgenerational effects of polychlorinated biphenyls: 2. Hypothalamic gene expression in rats. Biology of Reproduction 2021, 105(3):690-704.

96. Govorko D, Bekdash RA, Zhang C, Sarkar DK: Male germline transmits fetal alcohol adverse effect on hypothalamic proopiomelanocortin gene across generations. Biol Psychiatry 2012, 72(5):378-388.

97. Grandjean V, Yaman R, Cuzin F, Rassoulzadegan M: Inheritance of an epigenetic mark: The CpG DNA methyltransferase 1 is required for de novo establishment of a complex pattern of non-CpG methylation. PLoS ONE 2007, 2(11).

98. Graus-Nunes F, Dalla Corte Frantz E, Lannes WR, da Silva Menezes MC, Mandarim-de-Lacerda CA, Souza-Mello V: Pregestational maternal obesity impairs endocrine pancreas in male F1 and F2 progeny. Nutrition 2015, 31(2):380-387.

99. Grison S, Legendre A, Svilar L, Elie C, Kereselidze D, Gloaguen C, Lestaevel P, Martin JC, Souidi M: Multigenerational Exposure to Uranium Changes Sperm Metabolome in Rats. International Journal of Molecular Sciences 2022, 23(15).

100. Gross N, Taylor T, Crenshaw T, Khatib H: The Intergenerational Impacts of Paternal Diet on DNA Methylation and Offspring Phenotypes in Sheep. Frontiers in Genetics 2020, 11.

101. Guerrero-Bosagna C, Savenkova M, Haque MM, Nilsson E, Skinner MK: Environmentally Induced Epigenetic Transgenerational Inheritance of Altered Sertoli Cell Transcriptome and Epigenome: Molecular Etiology of Male Infertility. PLoS ONE 2013, 8(3).

102. Guerrero-Bosagna C, Settles M, Lucker B, Skinner MK: Epigenetic Transgenerational Actions of Vinclozolin on Promoter Regions of the Sperm Epigenome. PLOS ONE 2010, 5(9):e13100.

103. Guo Y, Bai D, Liu W, Liu Y, Zhang Y, Kou X, Chen J, Wang H, Teng X, Zuo J, Gao S: Altered sperm tsRNAs in aged male contribute to anxiety-like behavior in offspring. Aging Cell 2021, 20(9).

104. Guo ZJ, Xu D, Luo HW, Deng ZX, Zhong WH, Wang H: Prenatal nicotine exposure induces transgenerational neuroendocrine metabolic programming alteration in second-generation rats. Chin J Pharmacol Toxicol 2015, 29(2):277-283.

105. Guth LM, Ludlow AT, Witkowski S, Marshall MR, Lima LCJ, Venezia AC, Xiao T, Ting Lee ML, Spangenburg EE, Roth SM: Sex-specific effects of exercise ancestry on metabolic, morphological and gene expression phenotypes in multiple generations of mouse offspring. Experimental physiology 2013, 98(10):1469-1484.

106. Hanafi MY, Saleh MM, Saad MI, Abdelkhalek TM, Kamel MA: Transgenerational effects of obesity and malnourishment on diabetes risk in F2 generation. Molecular and Cellular Biochemistry 2016, 412(1-2):269-280.

107. Hao C, Gely-Pernot A, Kervarrec C, Boudjema M, Becker E, Khil P, Tevosian S, Jégou B, Smagulova F: Exposure to the widely used herbicide atrazine results in deregulation of global tissue-specific RNA transcription in the third generation and is associated with a global decrease of histone trimethylation in mice. Nucleic Acids Res 2016, 44(20):9784-9802.

108. Harasymowicz NS, Choi YR, Wu CL, Iannucci L, Tang R, Guilak F: Intergenerational Transmission of Diet-Induced Obesity, Metabolic Imbalance, and Osteoarthritis in Mice. Arthritis Rheum 2020, 72(4):632-644.

109. Harman JC, Guidry JJ, Gidday JM: Intermittent hypoxia promotes functional neuroprotection from retinal ischemia in untreated first-generation offspring: Proteomic mechanistic insights. Invest Ophthalmol Vis Sci 2020, 61(11).

110. Harper KM, Tunc-Ozcan E, Graf EN, Herzing LBK, Redei EE: Intergenerational and parent of origin effects of maternal calorie restriction on Igf2 expression in the adult rat hippocampus. Psychoneuroendocrinology 2014, 45:187-191.

111. Harper KM, Tunc-Ozcan E, Graf EN, Redei EE: Intergenerational effects of prenatal ethanol on glucose tolerance and insulin response. Physiological Genomics 2014, 46(5):159-168.

112. Hehar H, Ma I, Mychasiuk R: Intergenerational Transmission of Paternal Epigenetic Marks: Mechanisms Influencing Susceptibility to Post-Concussion Symptomology in a Rodent Model. Sci Rep 2017, 7(1).

113. Hernández-Rodríguez G, Zumbado M, Luzardo OP, Monterde JG, Blanco A, Boada LD: Multigenerational study of the hepatic effects exerted by the consumption of nonylphenol- and 4-octylphenol-contaminated drinking water in Sprague-Dawley rats. Environ Toxicol Pharmacol 2007, 23(1):73-81.

114. Hildebrandt MR, Germain DR, Monckton EA, Brun M, Godbout R: Ddx1 knockout results in transgenerational wild-type lethality in mice. Sci Rep 2015, 5.

115. Ho SM, Rao R, Ouyang B, Tam NNC, Schoch E, Song D, Ying J, Leung YK, Govindarajah V, Tarapore P: Three-generation study of male rats gestationally exposed to high butterfat and bisphenol a: Impaired spermatogenesis, penetrance with reduced severity. Nutrients 2021, 13(10).

116. Horan TS, Marre A, Hassold T, Lawson C, Hunt PA: Germline and reproductive tract effects intensify in male mice with successive generations of estrogenic exposure. PLoS Genetics 2017, 13(7).

117. Hsu PC, Li ZK, Lai CS, Tseng LH, Lee CW, Cheng FJ, Chang CY, Chen JR: Transgenerational effects of BDE-209 on male reproduction in F3 offspring rats. Chemosphere 2021, 272.

118. Hu H, Chen LB, Luo HW, Wu Y, Shen L, Kou H, Zhang L, Wang H: Transgenerational effects of prenatal ethanol ingestion-induced susceptibility to metabolic syndrome and the underlying mechanism in F2 rats. Chin J Pharmacol Toxicol 2014, 28(2):221-226.

119. Huerta-Cervantes M, Peña-Montes DJ, López-Vázquez MÁ, Montoya-Pérez R, Cortés-Rojo C, Olvera-Cortés ME, Saavedra-Molina A: Effects of gestational diabetes in cognitive behavior, oxidative stress and metabolism on the second-generation off-spring of rats. Nutrients 2021, 13(5).

120. Ismail A, Saliba Y, Fares N: Early Development of Cardiac Fibrosis in Young Old-Father Offspring. Oxidative Med Cell Longevity 2022, 2022.

121. Jašarević E, Hecht PM, Fritsche KL, Beversdorf DQ, Geary DC: Dissociable effects of dorsal and ventral hippocampal DHA content on spatial learning and anxiety-like behavior. Neurobiol Learn Mem 2014, 116:59-68.

122. Jenkins TG, James ER, Aston KI, Salas-Huetos A, Pastuszak AW, Smith KR, Hanson HA, Hotaling JM, Carrell DT: Age-associated sperm DNA methylation patterns do not directly persist trans-generationally. Epigenetics Chromatin 2019, 12(1).

123. Jia H, Morris CD, Williams RM, Loring JF, Thomas EA: HDAC inhibition imparts beneficial transgenerational effects in Huntington's disease mice via altered DNA and histone methylation. P Natl Acad Sci USA 2015, 112(1):E56-E64.

124. Jimenez-Chillaron JC, Isganaitis E, Charalambous M, Gesta S, Pentinat-Pelegrin T, Faucette RR, Otis JP, Chow A, Diaz R, Ferguson-Smith A, Patti ME: Intergenerational transmission of glucose intolerance and obesity by in utero undernutrition in mice. Diabetes 2009, 58(2):460-468.

125. Kaati G, Bygren LO, Edvinsson S: Cardiovascular and diabetes mortality determined by nutrition during parents' and grandparents' slow growth period. European Journal of Human Genetics 2002, 10(11):682-688.

126. Kaczmarek MM, Mendoza T, Kozak LP: Lactation undernutrition leads to multigenerational molecular programming of hypothalamic gene networks controlling reproduction. BMC Genomics 2016, 17(1).

127. Karahan G, Chan D, Shirane K, McClatchie T, Janssen S, Baltz JM, Lorincz M, Trasler J: Paternal MTHFR deficiency leads to hypomethylation of young retrotransposons and reproductive decline across two successive generations. Development (Cambridge) 2021, 148(13).

128. Karmakar PC, Ahn JS, Kim YH, Jung SE, Kim BJ, Lee HS, Kim SU, Rahman MS, Pang MG, Ryu BY: Paternal exposure to bisphenol-a transgenerationally impairs testis morphology, germ cell associations, and stemness properties of mouse spermatogonial stem cells. International Journal of Molecular Sciences 2020, 21(15):1-16.

129. Karmakar PC, Ahn JS, Kim YH, Jung SE, Kim BJ, Lee HS, Ryu BY: Gestational exposure to bisphenol a affects testicular morphology, germ cell associations, and functions of spermatogonial stem cells in male offspring. International Journal of Molecular Sciences 2020, 21(22):1-18.

130. Katzmarski N, Domínguez-Andrés J, Cirovic B, Renieris G, Ciarlo E, Le Roy D, Lepikhov K, Kattler K, Gasparoni G, Händler K et al: Transmission of trained immunity and heterologous resistance to infections across generations. Nature Immunology 2021, 22(11):1382-1390.

131. Kazachenka A, Bertozzi TM, Sjoberg-Herrera MK, Walker N, Gardner J, Gunning R, Pahita E, Adams S, Adams D, Ferguson-Smith AC: Identification, Characterization, and Heritability of Murine Metastable Epialleles: Implications for Non-genetic Inheritance. Cell 2018, 175(5):1259-1271.e1213.

132. Kempinas WG, Borges CS, Leite GAA, Figueiredo TM, Gregory M, Cyr DG: Prenatal exposure to betamethasone causes intergenerational impairment of epididymal development in the rat. Andrology 2019, 7(5):719-729.

133. King SE, Nilsson E, Beck D, Skinner MK: Adipocyte epigenetic alterations and potential therapeutic targets in transgenerationally inherited lean and obese phenotypes following ancestral exposures. Adipocyte 2019, 8(1):362-378.

134. Kiss D, Ambeskovic M, Montina T, Metz GAS: Stress transgenerationally programs metabolic pathways linked to altered mental health. Cellular and Molecular Life Sciences 2016, 73(23):4547-4557.

135. Kläver R, Sánchez V, Damm OS, Redmann K, Lahrmann E, Sandhowe-Klaverkamp R, Rohde C, Wistuba J, Ehmcke J, Schlatt S, Gromoll J: Direct but no transgenerational effects of decitabine and vorinostat on male fertility. PLoS ONE 2015, 10(2).

136. Klijs B, Angelini V, Mierau JO, Smidt N: The role of life-course socioeconomic and lifestyle factors in the intergenerational transmission of the metabolic syndrome: Results from the LifeLines Cohort Study. International Journal of Epidemiology 2016, 45(4):1236-1246.

137. Klukovich R, Nilsson E, Sadler-Riggleman I, Beck D, Xie Y, Yan W, Skinner MK: Environmental Toxicant Induced Epigenetic Transgenerational Inheritance of Prostate Pathology and Stromal-Epithelial Cell Epigenome and Transcriptome Alterations: Ancestral Origins of Prostate Disease. Sci Rep 2019, 9(1).

138. Ko CY, Wang SC, Liu YP: Sensorimotor gating deficits are inheritable in an isolation-rearing paradigm in rats. Behav Brain Res 2016, 302:115-121.

139. Koike T, Wakai T, Jincho Y, Sakashita A, Kobayashi H, Mizutani E, Wakayama S, Miura F, Ito T, Kono T: DNA methylation errors in cloned mouse sperm by germ line barrier evasion. Biology of Reproduction 2016, 94(6).

140. Kong QQ, Tian XD, Wang J, Yuan HJ, Ning SF, Luo MJ, Tan JH: A next-generation sequencing study on mechanisms by which restraint and social instability stresses of male mice alter offspring anxiety-like behavior. Sci Rep 2021, 11(1).

141. Le Corre L, Brulport A, Vaiman D, Chagnon MC: Epoxiconazole alters the histology and transcriptome of mouse liver in a transgenerational pattern. Chemico-Biological Interactions 2022, 360.

142. Le Q, Li Y, Hou W, Yan B, Yu X, Song H, Wang F, Ma L: Binge-like sucrose self-administration experience inhibits cocaine and sucrose seeking behavior in offspring. Front Behav Neurosci 2017, 11.

143. Legoff L, D'Cruz SC, Lebosq M, Gely-Pernot A, Bouchekhchoukha K, Monfort C, Kernanec PY, Tevosian S, Multigner L, Smagulova F: Developmental exposure to chlordecone induces transgenerational effects in somatic prostate tissue which are associated with epigenetic histone trimethylation changes. Environ Int 2021, 152.

144. Li CQ, Luo YW, Bi FF, Cui TT, Song L, Cao WY, Zhang JY, Li F, Xu JM, Hao W et al: Development of anxiety-like behavior via hippocampal IGF-2 signaling in the offspring of parental morphine exposure: Effect of enriched environment. Neuropsychopharmacology 2014, 39(12):2777-2787.

145. Li G, Chang H, Xia W, Mao Z, Li Y, Xu S: F0 maternal BPA exposure induced glucose intolerance of F2 generation through DNA methylation change in Gck. Toxicol Lett 2014, 228(3):192-199.

146. Li J, Liu S, Li S, Feng R, Na L, Chu X, Wu X, Niu Y, Sun Z, Han T et al: Prenatal exposure to famine and the development of hyperglycemia and type 2 diabetes in adulthood across consecutive generations: A population-based cohort study of families in Suihua, China. Am J Clin Nutr 2017, 105(1):221-227.

147. Li J, Yang Q, An R, Sesso HD, Zhong VW, Chan KHK, Madsen TE, Papandonatos GD, Zheng T, Wu WC et al: Famine and Trajectories of Body Mass Index, Waist Circumference, and Blood Pressure in Two Generations: Results from the CHNS from 1993-2015. Hypertension 2022, 79(3):518-531.

148. Li X, Shi X, Hou Y, Cao X, Gong L, Wang H, Li J, Li J, Wu C, Xiao D et al: Paternal hyperglycemia induces transgenerational inheritance of susceptibility to hepatic steatosis in rats involving altered methylation on Pparα promoter. Biochim Biophys Acta Mol Basis Dis 2019, 1865(1):147-160.

149. Li Z, Ma Y, Wang G, Wang H, Dai Y, Zhu Y, Chen S, Zheng X, Sun F: Overexpression of human-derived DNMT3A induced intergenerational inheritance of DNA methylation and gene expression variations in rat brain and testis. Epigenetics 2020, 15(10):1107-1120.

150. Lin C, Lin Y, Luo J, Yu J, Cheng Y, Wu X, Lin L, Lin Y: Maternal High-Fat Diet Multigenerationally Impairs Hippocampal Synaptic Plasticity and Memory in Male Rat Offspring. Endocrinology (United States) 2021, 162(1).

151. Lismer A, Dumeaux V, Lafleur C, Lambrot R, Brind'Amour J, Lorincz MC, Kimmins S: Histone H3 lysine 4 trimethylation in sperm is transmitted to the embryo and associated with diet-induced phenotypes in the offspring. Developmental Cell 2021, 56(5):671-686.e676.

152. Lismer A, Siklenka K, Lafleur C, Dumeaux V, Kimmins S: Sperm histone H3 lysine 4 trimethylation is altered in a genetic mouse model of transgenerational epigenetic inheritance. Nucleic Acids Res 2020, 48(20):11380-11393.

153. Liu J, Liao M, Huang R, You Y, Lin X, Yang H, Fan L, Zhong Y, Li X, Li J, Xiao X: Perinatal Combinational Exposure to Bisphenol A and a High-Fat Diet Contributes to Transgenerational Dysregulation of Cardiovascular and Metabolic Systems in Mice. Front Cell Dev Biol 2022, 10.

154. Long MT, Gurary EB, Massaro JM, Ma J, Hoffmann U, Chung RT, Benjamin EJ, Loomba R: Parental non-alcoholic fatty liver disease increases risk of non-alcoholic fatty liver disease in offspring. Liver Int 2019, 39(4):740-747.

155. Luo H, Deng Z, Liu L, Shen L, Kou H, He Z, Ping J, Xu D, Ma L, Chen L, Wang H: Prenatal caffeine ingestion induces transgenerational neuroendocrine metabolic programming alteration in second generation rats. Toxicology and Applied Pharmacology 2014, 274(3):383-392.

156. Ly L, Chan D, Aarabi M, Landry M, Behan NA, MacFarlane AJ, Trasler J: Intergenerational impact of paternal lifetime exposures to both folic acid deficiency and supplementation on reproductive outcomes and imprinted gene methylation. Mol Hum Reprod 2017, 23(7):461-477.

157. Ma J, Chen X, Liu Y, Xie Q, Sun Y, Chen J, Leng L, Yan H, Zhao B, Tang N: Ancestral TCDD exposure promotes epigenetic transgenerational inheritance of imprinted gene Igf2: Methylation status and DNMTs. Toxicology and Applied Pharmacology 2015, 289(2):193-202.

158. Madrid A, Borth LE, Hogan KJ, Hariharan N, Papale LA, Alisch RS, Iskandar BJ: DNA methylation and hydroxymethylation have distinct genome-wide profiles related to axonal regeneration. Epigenetics 2020:1-15.

159. Mahaq O, P. Rameli MA, Jaoi Edward M, Mohd Hanafi N, Abdul Aziz S, Abu Hassim H, Mohd Noor MH, Ahmad H: The effects of dietary edible bird nest supplementation on learning and memory functions of multigenerational mice. Brain Behav 2020, 10(11).

160. Manikkam M, Haque MM, Guerrero-Bosagna C, Nilsson EE, Skinner MK: Pesticide methoxychlor promotes the epigenetic transgenerational inheritance of adult-onset disease through the female germline. PLoS ONE 2014, 9(7).

161. Manners MT, Yohn NL, Lahens NF, Grant GR, Bartolomei MS, Blendy JA: Transgenerational inheritance of chronic adolescent stress: Effects of stress response and the amygdala transcriptome. Genes Brain Behav 2019, 18(7).

162. Mao Y, Zhao Y, Luo S, Chen H, Liu X, Wu T, Ding G, Liu X, Sheng J, Meng Y, Huang H: Advanced paternal age increased metabolic risks in mice offspring. Biochim Biophys Acta Mol Basis Dis 2022, 1868(5).

163. Martins Terra M, Schaeffer Fontoura T, Oliveira Nogueira A, Ferraz Lopes J, De Freitas Mathias PC, Andreazzi AE, De Oliveira Guerra M, Maria Peters V: Multigenerational effects of chronic maternal exposure to a high sugar/fat diet and physical training. J Dev Orig Health Dis 2020, 11(2):159-167.

164. Mashoodh R, Franks B, Curley JP, Champagne FA: Paternal social enrichment effects on maternal behavior and offspring growth. P Natl Acad Sci USA 2012, 109(SUPPL.2):17232-17238.

165. Master JS, Thouas GA, Harvey AJ, Sheedy JR, Hannan NJ, Gardner DK, Wlodek ME: Fathers that are born small program alterations in the next-generation preimplantation rat embryos. J Nutr 2015, 145(5):876-883.

166. Masuyama H, Mitsui T, Eguchi T, Tamada S, Hiramatsu Y: The effects of paternal high-fat diet exposure on offspring metabolism with epigenetic changes in the mouse adiponectin and leptin gene promoters. Am J Physiol Endocrinol Metab 2016, 311(1):E236-E245.

167. Mathai S, Derraik JGB, Cutfield WS, Dalziel SR, Harding JE, Biggs JB, Jefferies C, Hofman PL: Blood pressure abnormalities in adults born moderately preterm and their children. International Journal of Cardiology 2015, 181:152-154.

168. McBirney M, King SE, Pappalardo M, Houser E, Unkefer M, Nilsson E, Sadler-Riggleman I, Beck D, Winchester P, Skinner MK: Atrazine induced epigenetic transgenerational inheritance of disease, lean phenotype and sperm epimutation pathology biomarkers. PLoS ONE 2017, 12(9).

169. McCarthy DM, Lowe SE, Morgan TJ, Cannon EN, Biederman J, Spencer TJ, Bhide PG: Transgenerational transmission of behavioral phenotypes produced by exposure of male mice to saccharin and nicotine. Sci Rep 2020, 10(1).

170. McCarthy DM, Morgan TJ, Jr., Lowe SE, Williamson MJ, Spencer TJ, Biederman J, Bhide PG: Nicotine exposure of male mice produces behavioral impairment in multiple generations of descendants. PloS Biol 2018, 16(10).

171. McPherson NO, Fullston T, Bakos HW, Setchell BP, Lane M: Obese father’s metabolic state, adiposity, and reproductive capacity indicate son’s reproductive health. Fertil Steril 2014, 101(3):865-873.e861.

172. Mehta D, Pelzer ES, Bruenig D, Lawford B, McLeay S, Morris CP, Gibson JN, Young RM, Voisey J, Harvey W et al: DNA methylation from germline cells in veterans with PTSD. J Psychiatr Res 2019, 116:42-50.

173. Mehta D, Pelzer ES, Bruenig D, Lawford B, McLeay S, Morris CP, Gibson JN, Young RM, Voisey J, Harvey W et al: DNA methylation from germline cells in veterans with PTSD. J Psychiatr Res 2019, 116:42-50.

174. Mierzejewski P, Zakrzewska A, Kuczyńska J, Wyszogrodzka E, Dominiak M: Intergenerational implications of alcohol intake: metabolic disorders in alcohol-naïve rat offspring. PeerJ 2020, 8.

175. Moisiadis VG, Constantinof A, Kostaki A, Szyf M, Matthews SG: Prenatal Glucocorticoid Exposure Modifies Endocrine Function and Behaviour for 3 Generations Following Maternal and Paternal Transmission. Sci Rep 2017, 7(1).

176. Murashov AK, Pak ES, Koury M, Ajmera A, Jeyakumar M, Parker M, Williams O, Ding J, Walters D, Neufer PD: Paternal long-term exercise programs offspring for low energy expenditure and increased risk for obesity in mice. FASEB J 2016, 30(2):775-784.

177. Natale F, Spinelli M, Barbati SA, Leone L, Fusco S, Grassi C: High Fat Diet Multigenerationally Affects Hippocampal Neural Stem Cell Proliferation via Epigenetic Mechanisms. Cells 2022, 11(17).

178. Nilsson E, King SE, McBirney M, Kubsad D, Pappalardo M, Beck D, Sadler-Riggleman I, Skinner MK: Vinclozolin induced epigenetic transgenerational inheritance of pathologies and sperm epimutation biomarkers for specific diseases. PLoS ONE 2018, 13(8).

179. Nolan PB, Carrick-Ranson G, Stinear JW, Reading SA, Dalleck LC: Parent’s cardiorespiratory fitness, body mass, and chronic disease status is associated with metabolic syndrome in young adults: A preliminary study. Int J Environ Res Public Health 2019, 16(10).

180. Northstone K, Golding J, Davey Smith G, Miller LL, Pembrey M: Prepubertal start of father’s smoking and increased body fat in his sons: further characterisation of paternal transgenerational responses. European Journal of Human Genetics 2014, 22(12):1382-1386.

181. O'Brien EA, Ensbey KS, Day BW, Baldock PA, Barry G: Direct evidence for transport of RNA from the mouse brain to the germline and offspring. BMC Biol 2020, 18(1).

182. Ojo Alese O, Mabandla MV: Transgenerational deep sequencing revealed hypermethylation of hippocampal mGluR1 gene with altered mRNA expression of mGluR5 and mGluR3 associated with behavioral changes in Sprague Dawley rats with history of prolonged febrile seizure. PLoS ONE 2019, 14(11).

183. Orozco LD, Rubbi L, Martin LJ, Fang F, Hormozdiari F, Che N, Smith AD, Lusis AJ, Pellegrini M: Intergenerational genomic DNA methylation patterns in mouse hybrid strains. Genome biology 2014, 15(5):R68.

184. Pachenari N, Azizi H, Ghasemi E, Azadi M, Semnanian S: Exposure to opiates in male adolescent rats alters pain perception in the male offspring. Behav Pharmacol 2018, 29:255-260.

185. Padmanabhan N, Rakoczy J, Kondratowicz M, Menelaou K, Blake GET, Watson ED: Multigenerational analysis of sex-specific phenotypic differences at midgestation caused by abnormal folate metabolism. Environmental Epigenetics 2017, 3(4).

186. Painter RC, Osmond C, Gluckman P, Hanson M, Phillips DIW, Roseboom TJ: Transgenerational effects of prenatal exposure to the Dutch famine on neonatal adiposity and health in later life. BJOG Int J Obstet Gynaecol 2008, 115(10):1243-1249.

187. Park YJ, Herman H, Gao Y, Lindroth AM, Hu BY, Murphy PJ, Putnam JR, Soloway PD: Sequences sufficient for programming imprinted germline dna methylation defined. PLoS ONE 2012, 7(3).

188. Pentinat T, Ramon-Krauel M, Cebria J, Diaz R, Jimenez-Chillaron JC: Transgenerational inheritance of glucose intolerance in a mouse model of neonatal overnutrition. Endocrinology 2010, 151(12):5617-5623.

189. Pepin AS, Lafleur C, Lambrot R, Dumeaux V, Kimmins S: Sperm histone H3 lysine 4 tri-methylation serves as a metabolic sensor of paternal obesity and is associated with the inheritance of metabolic dysfunction. Mol Metab 2022, 59.

190. Pinel A, Rigaudière JP, Jouve C, Montaurier C, Jousse C, Lhomme M, Morio B, Capel F: Transgenerational supplementation with eicosapentaenoic acid reduced the metabolic consequences on the whole body and skeletal muscle in mice receiving an obesogenic diet. European Journal of Nutrition 2021, 60(6):3143-3157.

191. Polat S, Caner A: Transgenerational impact of topical steroid application on superoxide dismutase activities of hypothalamus-pituitary-adrenal axis in rats. Can J Physiol Pharmacol 2022, 100(5):386-392.

192. Popoola DO, Nizhnikov ME, Cameron NM: Strain-specific programming of prenatal ethanol exposure across generations. Alcohol 2017, 60:191-199.

193. Quinnies KM, Harris EP, Snyder RW, Sumner SS, Rissman EF: Direct and transgenerational effects of low doses of perinatal Di-(2-ethylhexyl) phthalate (DEHP) on social behaviors in mice. PLoS ONE 2017, 12(2).

194. Raad G, Serra F, Martin L, Derieppe M-A, Gilleron J, Costa VL, Pisani DF, Amri E-Z, Trabucchi M, Grandjean V: Paternal multigenerational exposure to an obesogenic diet drives epigenetic predisposition to metabolic diseases in mice. eLife 2021, 10.

195. Raad G, Serra F, Martin L, Derieppe MA, Gilleron J, Costa VL, Pisani DF, Amri EZ, Trabucchi M, Grandjean V: Paternal multigenerational exposure to an obesogenic diet drives epigenetic predisposition to metabolic diseases in mice. eLife 2021, 10.

196. Rahman MS, Pang W-K, Ryu D-Y, Park Y-J, Ryu B-Y, Pang M-G: Multigenerational impacts of gestational bisphenol A exposure on the sperm function and fertility of male mice. Journal of Hazardous Materials 2021, 416:125791.

197. Rakyan VK, Chong S, Champ ME, Cuthbert PC, Morgan HD, Luu KVK, Whitelaw E: Transgenerational inheritance of epigenetic states at the murine AxinFu allele occurs after maternal and paternal transmission. P Natl Acad Sci USA 2003, 100(5):2538-2543.

198. Rawat A, Guo J, Renoir T, Pang TY, Hannan AJ: Hypersensitivity to sertraline in the absence of hippocampal 5-HT1AR and 5-HTT gene expression changes following paternal corticosterone treatment. Environmental Epigenetics 2018, 4(2).

199. Razoux F, Russig H, Mueggler T, Baltes C, Dikaiou K, Rudin M, Mansuy IM: Transgenerational disruption of functional 5-HT 1A R-induced connectivity in the adult mouse brain by traumatic stress in early life. Mol Psychiatry 2017, 22(4):519-526.

200. Ren J, Cheng Y, Ming ZH, Dong XY, Zhou YZ, Ding GL, Pang HY, Rahman TU, Akbar R, Huang HF, Sheng JZ: Intrauterine hyperglycemia exposure results in intergenerational inheritance via DNA methylation reprogramming on F1 PGCs. Epigenetics Chromatin 2018, 11(1).

201. Risal S, Manti M, Lu H, Fornes R, Larsson H, Benrick A, Deng Q, Cesta CE, Rosenqvist MA, Stener-Victorin E: Prenatal androgen exposure causes a sexually dimorphic transgenerational increase in offspring susceptibility to anxiety disorders. Transl Psychiatry 2021, 11(1).

202. Riyahi J, Abdoli B, Gelfo F, Petrosini L, Khatami L, Meftahi GH, Haghparast A: Multigenerational effects of paternal spatial training are lasting in the F1 and F2 male offspring. Behav Pharmacol 2022, 33(5):342-354.

203. Riyahi J, Abdoli B, Haghparast A, Petrosini L: Intergenerational effect of parental spatial training on offspring learning: Evidence for sex differences in memory function. Brain Res Bull 2019, 153:314-323.

204. Rodgers AB, Morgan CP, Leu NA, Bale TL: Transgenerational epigenetic programming via sperm microRNA recapitulates effects of paternal stress. P Natl Acad Sci USA 2015, 112(44):13699-13704.

205. Romano KA, Martinez-del Campo A, Kasahara K, Chittim CL, Vivas EI, Amador-Noguez D, Balskus EP, Rey FE: Metabolic, Epigenetic, and Transgenerational Effects of Gut Bacterial Choline Consumption. Cell Host and Microbe 2017, 22(3):279-290.e277.

206. Rompala GR, Finegersh A, Homanics GE: Paternal preconception ethanol exposure blunts hypothalamic-pituitary-adrenal axis responsivity and stress-induced excessive fluid intake in male mice. Alcohol 2016, 53:19-25.

207. Rompala GR, Finegersh A, Slater M, Homanics GE: Paternal preconception alcohol exposure imparts intergenerational alcohol-related behaviors to male offspring on a pure C57BL/6J background. Alcohol 2017, 60:169-177.

208. Sabzevari S, Rohbani K, Sadat-Shirazi MS, Babhadi-Ashar N, Shakeri A, Ashabi G, Khalifeh S, Ale-Ebrahim M, Zarrindast MR: Morphine exposure before conception affects anxiety-like behavior and CRF level (in the CSF and plasma) in the adult male offspring. Brain Res Bull 2019, 144:122-131.

209. Sadler-Riggleman I, Klukovich R, Nilsson E, Beck D, Xie Y, Yan W, Skinner MK: Epigenetic transgenerational inheritance of testis pathology and Sertoli cell epimutations: Generational origins of male infertility. Environmental Epigenetics 2019, 5(3).

210. Saidur Rahman MD, Pang WK, Ryu DY, Park YJ, Pang MG: Multigenerational and transgenerational impact of paternal bisphenol A exposure on male fertility in a mouse model. Hum Reprod 2020, 35(8):1740-1752.

211. Sakamoto M, Ito D, Inoue R, Wakayama S, Kikuchi Y, Yang L, Hayashi E, Emura R, Shiura H, Kohda T et al: Paternally inherited H3K27me3 affects chromatin accessibility in mouse embryos produced by round spermatid injection. Development (Cambridge) 2022, 149(18).

212. Sanchez-Garrido MA, Ruiz-Pino F, Velasco I, Barroso A, Fernandois D, Heras V, Manfredi-Lozano M, Vazquez MJ, Castellano JM, Roa J et al: Intergenerational Influence of paternal obesity on metabolic and reproductive health parameters of the offspring: Male-preferential impact and involvement of kiss1-mediated pathways. Endocrinology 2018, 159(2):1005-1018.

213. Sarker G, Berrens R, von Arx J, Pelczar P, Reik W, Wolfrum C, Peleg-Raibstein D: Transgenerational transmission of hedonic behaviors and metabolic phenotypes induced by maternal overnutrition. Transl Psychiatry 2018, 8(1).

214. Schellong K, Melchior K, Ziska T, Rancourt RC, Henrich W, Plagemann A: Maternal but not paternal high-fat diet (HFD) exposure at conception predisposes for ‘diabesity’ in offspring generations. Int J Environ Res Public Health 2020, 17(12):1-14.

215. Seong HY, Cho HM, Kim M, Kim I: Maternal high-fructose intake induces multigenerational activation of the renin-angiotensin-aldosterone system. Hypertension 2019, 74(3):518-525.

216. Sharma A: Transcriptomic data reanalysis allows for a contribution of embryonic transcriptional change-induced gene expression reprogramming in transgenerational epigenetic inheritance. Environmental Epigenetics 2016, 2(2).

217. Sheth VG, Sharma N, Kabeer SW, Tikoo K: Lactobacillus rhamnosus supplementation ameliorates high fat diet-induced epigenetic alterations and prevents its intergenerational inheritance. Life Sciences 2022, 311.

218. Shi M, Whorton AE, Sekulovski N, Maclean JA, Hayashi K: Prenatal Exposure to Bisphenol A, E, and S Induces Transgenerational Effects on Male Reproductive Functions in Mice. Toxicological Sciences 2019, 172(2):303-315.

219. Shi X, Li X, Hou Y, Cao X, Zhang Y, Wang H, Wang H, Peng C, Li J, Li Q et al: Paternal hyperglycemia in rats exacerbates the development of obesity in offspring. Journal of Endocrinology 2017, 234(2):175-186.

220. Short AK, Fennell KA, Perreau VM, Fox A, O'Bryan MK, Kim JH, Bredy TW, Pang TY, Hannan AJ: Elevated paternal glucocorticoid exposure alters the small noncoding RNA profile in sperm and modifies anxiety and depressive phenotypes in the offspring. Transl Psychiatry 2016, 6(6).

221. Short AK, Yeshurun S, Powell R, Perreau VM, Fox A, Kim JH, Pang TY, Hannan AJ: Exercise alters mouse sperm small noncoding RNAs and induces a transgenerational modification of male offspring conditioned fear and anxiety. Transl Psychiatry 2017, 7(5).

222. Siklenka K, Erkek S, Godmann M, Lambrot R, McGraw S, Lafleur C, Cohen T, Xia J, Suderman M, Hallett M et al: Disruption of histone methylation in developing sperm impairs offspring health transgenerationally. Science 2015, 350(6261):aab2006.

223. Skinner MK, Anway MD, Savenkova MI, Gore AC, Crews D: Transgenerational epigenetic programming of the brain transcriptome and anxiety behavior. PLoS ONE 2008, 3(11).

224. Skinner MK, Ben Maamar M, Sadler-Riggleman I, Beck D, Nilsson E, McBirney M, Klukovich R, Xie Y, Tang C, Yan W: Alterations in sperm DNA methylation, non-coding RNA and histone retention associate with DDT-induced epigenetic transgenerational inheritance of disease. Epigenetics & Chromatin 2018, 11(1):8.

225. Skinner MK, Manikkam M, Tracey R, Guerrero-Bosagna C, Haque M, Nilsson EE: Ancestral dichlorodiphenyltrichloroethane (DDT) exposure promotes epigenetic transgenerational inheritance of obesity. BMC Medicine 2013, 11(1):228.

226. Skinner MK, Mohan M, Haque MM, Zhang B, Savenkova MI: Epigenetic transgenerational inheritance of somatic transcriptomes and epigenetic controlregions. Genome biology 2012, 13(10).

227. Skinner MK, Nilsson E, Sadler-Riggleman I, Beck D, Ben Maamar M, McCarrey JR: Transgenerational sperm DNA methylation epimutation developmental origins following ancestral vinclozolin exposure. Epigenetics 2019, 14(7):721-739.

228. Skinner MK, Savenkova MI, Zhang B, Gore AC, Crews D: Gene bionetworks involved in the epigenetic transgenerational inheritance of altered mate preference: Environmental epigenetics and evolutionary biology. BMC Genomics 2014, 15(1).

229. Sobolewski M, Abston K, Conrad K, Marvin E, Harvey K, Susiarjo M, Cory-Slechta DA: Lineage-and sex-dependent behavioral and biochemical transgenerational consequences of developmental exposure to lead, prenatal stress, and combined lead and prenatal stress in mice. Environ Health Perspect 2020, 128(2).

230. Song Y, Wu N, Wang S, Gao M, Song P, Lou J, Tan Y, Liu K: Transgenerational impaired male fertility with an Igf2 epigenetic defect in the rat are induced by the endocrine disruptor p,p′-DDE. Hum Reprod 2014, 29(11):2512-2521.

231. Song Y, Yang L: Transgenerational pancreatic impairment with Igf2/H19 epigenetic alteration induced by p,p’-DDE exposure in early life. Toxicol Lett 2017, 280:222-231.

232. Song Y, Yang L: Transgenerational impaired spermatogenesis with sperm H19 and Gtl2 hypomethylation induced by the endocrine disruptor p,p’-DDE. Toxicol Lett 2018, 297:34-41.

233. Sosnina SF, Okatenko PV, Sokolnikov ME: Consequences of parental preconceptional irradiation: Endocrine-metabolic pathology in offspring. Radiatsionnaya Gygiena 2022, 15(4):15-33.

234. Sousa Neto IVD, Tibana RA, Silva LGDOD, Lira EMD, Prado GPGD, Almeida JAD, Franco OL, Durigan JLQ, Adesida AB, Sousa MVD et al: Paternal Resistance Training Modulates Calcaneal Tendon Proteome in the Offspring Exposed to High-Fat Diet. Front Cell Dev Biol 2020, 8.

235. Souza TL, Batschauer AR, Brito PM, Oliveira Ribeire CA, Martino-Andrade AJ, Ortolani-Machado CF: Multigenerational analysis of the functional status of male reproductive system in mice after exposure to realistic doses of manganese. Food and Chemical Toxicology 2019, 133.

236. Stringer JM, Forster SC, Qu Z, Prokopuk L, O'Bryan MK, Gardner DK, White SJ, Adelson D, Western PS: Reduced PRC2 function alters male germline epigenetic programming and paternal inheritance. BMC Biol 2018, 16(1).

237. Suen JL, Wu TT, Li YH, Lee CL, Kuo FC, Yan PS, Wu CF, Tran M, Wang CJ, Hung CH et al: Environmental Factor-Mediated Transgenerational Inheritance of Igf2r Hypomethylation and Pulmonary Allergic Response via Targeting Dendritic Cells. Front Immunol 2020, 11.

238. Swinford-Jackson SE, Fant B, Wimmer ME, Chan D, Knouse MC, Sarmiento M, Thomas AS, Huffman PJ, Mankame S, Worobey SJ, Christopher Pierce R: Cocaine-Induced Changes in Sperm Cdkn1a Methylation Are Associated with Cocaine Resistance in Male Offspring. Journal of Neuroscience 2022, 42(14):2905-2916.

239. Tait AH, Raubenheimer D, Green MP, Cupido CL, Gluckman PD, Vickers MH: Successive generations in a rat model respond differently to a constant obesogenic environment. PLoS ONE 2015, 10(7).

240. Tang A, Huang Y, Li Z, Wan S, Mou L, Yin G, Li N, Xie J, Xia Y, Li X et al: Analysis of a four generation family reveals the widespread sequence-dependent maintenance of allelic DNA methylation in somatic and germ cells. Sci Rep 2016, 6.

241. Tang Q, Tu B, Jiang X, Zhang J, Bai L, Meng P, Zhang L, Qin X, Wang B, Chen C, Zou Z: Exposure to carbon black nanoparticles during pregnancy aggravates lipopolysaccharide-induced lung injury in offspring: An intergenerational effect. Am J Physiol Lung Cell Mol Physiol 2021, 321(5):L900-L911.

242. Terashima M, Barbour S, Ren J, Yu W, Han Y, Muegge K: Effect of high fat diet on paternal sperm histone distribution and male offspring liver gene expression. Epigenetics 2015, 10(9):861-871.

243. Thorson JLM, Beck D, Maamar MB, Nilsson EE, McBirney M, Skinner MK: Epigenome-wide association study for atrazine induced transgenerational DNA methylation and histone retention sperm epigenetic biomarkers for disease. PLoS ONE 2020, 15(12 December).

244. Toschke AM, Ehlin A, Koletzko B, Montgomery SM: Paternal smoking is associated with a decreased prevalence of type 1 diabetes mellitus among offspring in two national British birth cohort studies (NCDS and BCS70). J Perinat Med 2007, 35(1):43-47.

245. Toussaint AB, Foster W, Jones JM, Kaufmann S, Wachira M, Hughes R, Bongiovanni AR, Famularo ST, Dunham BP, Schwark R et al: Chronic paternal morphine exposure increases sensitivity to morphine-derived pain relief in male progeny. Sci Adv 2022, 8(7).

246. Tunc-Ozcan E, Harper KM, Graf EN, Redei EE: Thyroxine administration prevents matrilineal intergenerational consequences of in utero ethanol exposure in rats. Hormones and behavior 2016, 82:1-10.

247. Tyebji S, Hannan AJ, Tonkin CJ: Pathogenic Infection in Male Mice Changes Sperm Small RNA Profiles and Transgenerationally Alters Offspring Behavior. Cell Reports 2020, 31(4).

248. Van De Werken C, Van Der Heijden GW, Eleveld C, Teeuwssen M, Albert M, Baarends WM, Laven JSE, Peters AHFM, Baart EB: Paternal heterochromatin formation in human embryos is H3K9/HP1 directed and primed by sperm-derived histone modifications. Nat commun 2014, 5.

249. Van Steenwyk G, Roszkowski M, Manuella F, Franklin TB, Mansuy IM: Transgenerational inheritance of behavioral and metabolic effects of paternal exposure to traumatic stress in early postnatal life: Evidence in the 4th generation. Environmental Epigenetics 2018, 4(2):1-8.

250. Vassoler FM, Toorie AM, Teceno DN, Walia P, Moore DJ, Patton TD, Byrnes EM: Paternal morphine exposure induces bidirectional effects on cocaine versus opioid self-administration. Neuropharmacology 2020, 162.

251. Vaughan OR, Phillips HM, Everden AJ, Sferruzzi-Perri AN, Fowden AL: Dexamethasone treatment of pregnant F0 mice leads to parent of origin-specific changes in placental function of the F2 generation. Reproduction, Fertility and Development 2015, 27(4):704-711.

252. Veenendaal MVE, Painter RC, De Rooij SR, Bossuyt PMM, Van Der Post JAM, Gluckman PD, Hanson MA, Roseboom TJ: Transgenerational effects of prenatal exposure to the 1944-45 Dutch famine. BJOG Int J Obstet Gynaecol 2013, 120(5):548-554.

253. Velazquez R, Ferreira E, Winslow W, Dave N, Piras IS, Naymik M, Huentelman MJ, Tran A, Caccamo A, Oddo S: Maternal choline supplementation ameliorates Alzheimer’s disease pathology by reducing brain homocysteine levels across multiple generations. Mol Psychiatry 2020, 25(10):2620-2629.

254. Walker DI, Marder ME, Yano Y, Terrell M, Liang Y, Barr DB, Miller GW, Jones DP, Marcus M, Pennell KD: Multigenerational metabolic profiling in the Michigan PBB registry. Environ Res 2019, 172:182-193.

255. Wang B, Xia L, Zhu D, Zeng H, Wei B, Lu L, Li W, Shi Y, Liu J, Zhang Y, Sun M: Paternal High-Fat Diet Altered Sperm 5'tsRNA-Gly-GCC Is Associated With Enhanced Gluconeogenesis in the Offspring. Front Mol Biosci 2022, 9.

256. Wang HLV, Forestier S, Corces VG: Exposure to sevoflurane results in changes of transcription factor occupancy in sperm and inheritance of autism. Biology of Reproduction 2021, 105(3):705-719.

257. Wei Y, Yang CR, Wei YP, Zhao ZA, Hou Y, Schatten H, Sun QY: Paternally induced transgenerational inheritance of susceptibility to diabetes in mammals. P Natl Acad Sci USA 2014, 111(5):1873-1878.

258. Wen Y, Rattan S, Flaws JA, Irudayaraj J: Multi and transgenerational epigenetic effects of di-(2-ethylhexyl) phthalate (DEHP) in liver. Toxicology and Applied Pharmacology 2020, 402.

259. Weyrich A, Lenz D, Jeschek M, Chung TH, Rübensam K, Göritz F, Jewgenow K, Fickel J: Paternal intergenerational epigenetic response to heat exposure in male Wild Guinea pigs. Mol Ecol 2016, 25(8):1729-1740.

260. Wijnands KPJ, Obermann-Borst SA, Sijbrands EJG, Wildhagen MF, Helbing WA, Steegers-Theunissen RPM: Cardiovascular diseases in grandparents and the risk of congenital heart diseases in grandchildren. J Dev Orig Health Dis 2014, 5(2):152-158.

261. Williams C, Suderman M, Guggenheim JA, Ellis G, Gregory S, Iles-Caven Y, Northstone K, Golding J, Pembrey M: Grandmothers’ smoking in pregnancy is associated with a reduced prevalence of early-onset myopia. Sci Rep 2019, 9(1).

262. Wolstenholme JT, Drobná Z, Henriksen AD, Goldsby JA, Stevenson R, Irvin JW, Flaws JA, Rissman EF: Transgenerational Bisphenol A Causes Deficits in Social Recognition and Alters Postsynaptic Density Genes in Mice. Endocrinology 2019, 160(8):1854-1867.

263. Wu HY, Cheng Y, Jin LY, Zhou Y, Pang HY, Zhu H, Yan CC, Yan YS, Yu JE, Sheng JZ, Huang HF: Paternal obesity impairs hepatic gluconeogenesis of offspring by altering Igf2/H19 DNA methylation. Mol Cell Endocrinol 2021, 529.

264. Wu L, Lu Y, Jiao Y, Liu B, Li S, Li Y, Xing F, Chen D, Liu X, Zhao J et al: Paternal Psychological Stress Reprograms Hepatic Gluconeogenesis in Offspring. Cell Metab 2016, 23(4):735-743.

265. Wu R, Zhang H, Xue W, Zou Z, Lu C, Xia B, Wang W, Chen G: Transgenerational impairment of hippocampal Akt-mTOR signaling and behavioral deficits in the offspring of mice that experience postpartum depression-like illness. Prog Neuro-Psychopharmacol Biol Psychiatry 2017, 73:11-18.

266. Wyck S, Herrera C, Requena CE, Bittner L, Hajkova P, Bollwein H, Santoro R: Oxidative stress in sperm affects the epigenetic reprogramming in early embryonic development. Epigenetics Chromatin 2018, 11(1).

267. Xia D, Parvizi N, Zhou Y, Xu K, Jiang H, Li R, Hang Y, Lu Y: Paternal fenvalerate exposure influences reproductive functions in the offspring. Reproductive Sciences 2013, 20(11):1308-1315.

268. Xie K, Ryan DP, Pearson BL, Henzel KS, Neff F, Vidal RO, Hennion M, Lehmann I, Schleif M, Schröder S et al: Epigenetic alterations in longevity regulators, reduced life span, and exacerbated aging-related pathology in old father offspring mice. P Natl Acad Sci USA 2018, 115(10):E2348-E2357.

269. Xu N, Lei L, Lin Y, Ju LS, Morey TE, Gravenstein N, Yang J, Martynyuk AE: A Methyltransferase Inhibitor (Decitabine) Alleviates Intergenerational Effects of Paternal Neonatal Exposure to Anesthesia with Sevoflurane. Anesth Analg 2020:1291-1299.

270. Xu Q, Hu L, Chen S, Fu X, Gong P, Huang Z, Miao W, Jin C, Jin Y: Parental exposure 3-methylcholanthrene disturbed the enterohepatic circulation in F1 generation of mice. Chemosphere 2022, 286.

271. Yang Y, Yang S, Jia Y, Yin C, Zhao R: Sex-biased transgenerational transmission of betaine-induced epigenetic modifications in glucocorticoid receptor gene and its down-stream BDNF/ERK pathway in rat hippocampus. Nutr Neurosci 2022, 25(4):746-757.

272. Yen AMF, Boucher BJ, Chiu SYH, Fann JCY, Chen SLS, Huang KC, Chen HH: Longer duration and earlier age of onset of paternal betel chewing and smoking increase metabolic syndrome risk in human offspring, independently, in a community-based screening program in Taiwan. Circulation 2016, 134(5):392-404.

273. Yeshurun S, Rogers J, Short AK, Renoir T, Pang TY, Hannan AJ: Elevated paternal glucocorticoid exposure modifies memory retention in female offspring. Psychoneuroendocrinology 2017, 83:9-18.

274. Yoshida K, Maekawa T, Ly NH, Fujita SI, Muratani M, Ando M, Katou Y, Araki H, Miura F, Shirahige K et al: ATF7-Dependent Epigenetic Changes Are Required for the Intergenerational Effect of a Paternal Low-Protein Diet. Molecular Cell 2020, 78(3):445-458.e446.

275. Yoshizaki K, Kimura R, Kobayashi H, Oki S, Kikkawa T, Mai L, Koike K, Mochizuki K, Inada H, Matsui Y et al: Paternal age affects offspring via an epigenetic mechanism involving REST/NRSF. EMBO Rep 2021, 22(2).

276. Youngson N, Lecomte V, Maloney C, Leung P, Liu J, Hesson L, Luciani F, Krause L, Morris M: Obesity-induced sperm DNA methylation changes at satellite repeats are reprogrammed in rat offspring. Asian Journal of Andrology 2016, 18(6):930-936.

277. Yuan B, Wu W, Chen M, Gu H, Tang Q, Guo D, Chen T, Chen Y, Lu C, Song L et al: Metabolomics Reveals a Role of Betaine in Prenatal DBP Exposure-Induced Epigenetic Transgenerational Failure of Spermatogenesis in Rats. Toxicological Sciences 2017, 158(2):356-366.

278. Yuan S, Oliver D, Schuster A, Zheng H, Yan W: Breeding scheme and maternal small RNAs affect the efficiency of transgenerational inheritance of a paramutation in mice. Sci Rep 2015, 5.

279. Zaidan H, Ramaswami G, Golumbic YN, Sher N, Malik A, Barak M, Galiani D, Dekel N, Li JB, Gaisler-Salomon I: A-to-I RNA editing in the rat brain is age-dependent, region-specific and sensitive to environmental stress across generations. BMC Genomics 2018, 19(1).

280. Zatecka E, Bohuslavova R, Valaskova E, Margaryan H, Elzeinova F, Kubatova A, Hylmarova S, Peknicova J, Pavlinkova G: The Transgenerational Transmission of the Paternal Type 2 Diabetes-Induced Subfertility Phenotype. Front Endocrinol 2021, 12.

281. Zeid D, Goldberg LR, Seemiller LR, Mooney-Leber S, Smith PB, Gould TJ: Multigenerational nicotine exposure affects offspring nicotine metabolism, nicotine-induced hypothermia, and basal corticosterone in a sex-dependent manner. Neurotoxicol Teratol 2021, 85.

282. Zeng L, Zhou J, Zhang Y, Wang X, Wang M, Su P: Differential Expression Profiles and Potential Intergenerational Functions of tRNA-Derived Small RNAs in Mice After Cadmium Exposure. Front Cell Dev Biol 2022, 9.

283. Zeybel M, Hardy T, Wong YK, Mathers JC, Fox CR, Gackowska A, Oakley F, Burt AD, Wilson CL, Anstee QM et al: Multigenerational epigenetic adaptation of the hepatic wound-healing response. Nature Medicine 2012, 18(9):1369-1377.

284. Zhang D, Dai J, Zhang M, Xie Y, Cao Y, He G, Xu W, Wang L, Qiao Z, Qiao Z: Paternal nicotine exposure promotes hepatic fibrosis in offspring. Toxicol Lett 2021, 343:44-55.

285. Zhang H, Shan L, Aniagu S, Jiang Y, Chen T: Paternal acrylamide exposure induces transgenerational effects on sperm parameters and learning capability in mice. Food and Chemical Toxicology 2022, 161.

286. Zhang HL, Yi M, Li D, Li R, Zhao Y, Qiao J: Transgenerational Inheritance of Reproductive and Metabolic Phenotypes in PCOS Rats. Front Endocrinol 2020, 11.

287. Zhang J, Yao Y, Pan J, Guo X, Han X, Zhou J, Meng X: Maternal exposure to Di-(2-ethylhexyl) phthalate (DEHP) activates the PI3K/Akt/mTOR signaling pathway in F1 and F2 generation adult mouse testis. Experimental Cell Research 2020, 394(2).

288. Zhang S, Li X, Wang Z, Liu Y, Gao Y, Tan L, Liu E, Zhou Q, Xu C, Wang X et al: Paternal spatial training enhances offspring's cognitive performance and synaptic plasticity in wild-type but not improve memory deficit in Alzheimer's mice. Sci Rep 2017, 7(1).

289. Zhang Y, Zhang X, Shi J, Tuorto F, Li X, Liu Y, Liebers R, Zhang L, Qu Y, Qian J et al: Dnmt2 mediates intergenerational transmission of paternally acquired metabolic disorders through sperm small non-coding RNAs. Nature Cell Biology 2018, 20(5):535-540.

290. Zhang Z, Li N, Chen R, Lee T, Gao Y, Yuan Z, Nie Y, Sun T: Prenatal stress leads to deficits in brain development, mood related behaviors and gut microbiota in offspring. Neurobiol Stress 2021, 15.

291. Zhang Z, Luo X, Lv Y, Yan L, Xu S, Wang Y, Zhong Y, Hang C, Jyotsnav J, Lai D et al: Intrauterine growth restriction programs intergenerational transmission of pulmonary arterial hypertension and endothelial dysfunction via sperm epigenetic modifications. Hypertension 2019, 74(5):1160-1171.

292. Zhao WL, Gu NH, Li ZZ, Wang GS, Cheng CY, Sun F: Autism-like behaviors and abnormality of glucose metabolism in offspring derived from aging males with epigenetically modified sperm. Aging 2020, 12(19):19766-19784.

293. Zheng X, Li Z, Wang G, Wang H, Zhou Y, Zhao X, Cheng CY, Qiao Y, Sun F: Sperm epigenetic alterations contribute to inter- and transgenerational effects of paternal exposure to long-term psychological stress via evading offspring embryonic reprogramming. Cell Discov 2021, 7(1).

294. Zhou Y, Zhu H, Wu HY, Jin LY, Chen B, Pang HY, Ming ZH, Cheng Y, Zhou CL, Guo MX et al: Diet-Induced Paternal Obesity Impairs Cognitive Function in Offspring by Mediating Epigenetic Modifications in Spermatozoa. Obesity 2018, 26(11):1749-1757.

295. Zulyniak MA, Fuller H, Iles MM: Investigation of the Causal Association between Long-Chain n-6 Polyunsaturated Fatty Acid Synthesis and the Risk of Type 2 Diabetes: A Mendelian Randomization Analysis. Lifestyle Genomics 2020, 13(5):146-153.
